# Supplementary material for: Ex vivo expansion of hematopoietic stem and progenitor cells from human mobilized peripheral blood for gene therapy applications
Source: Mol Ther. 2026 May 26;34(8):4581–603. doi: 10.1016/j.ymthe.2026.05.014 (PMC13464310; doi:10.1016/j.ymthe.2026.05.014)
Supplement: Supplementary file 1 [file mmc1.pdf]

YMTHE, Volume *34*

Supplemental Information

Ex vivo expansion of hematopoietic stem and progenitor cells from human mobilized peripheral blood for gene therapy applications

Erika Zonari, Matteo Maria Naldini, Matteo Barcella, Monica Volpin, Francesca Vincenti, Giacomo Desantis, Leila Hadadi, Carolina Caserta, Ilaria Galasso, Beatrice Martini, Federico Midena, Giacomo Farina, Roberta Vacca, Francesca Tucci, Leonardo Ormoli, Ilaria Visigalli, Michela Vezzoli, Dejan Lazarevic, Ivan Merelli, Stephanie Z. Xie, John E. Dick, Raffaella Di Micco, Eugenio Montini, and Bernhard Gentner

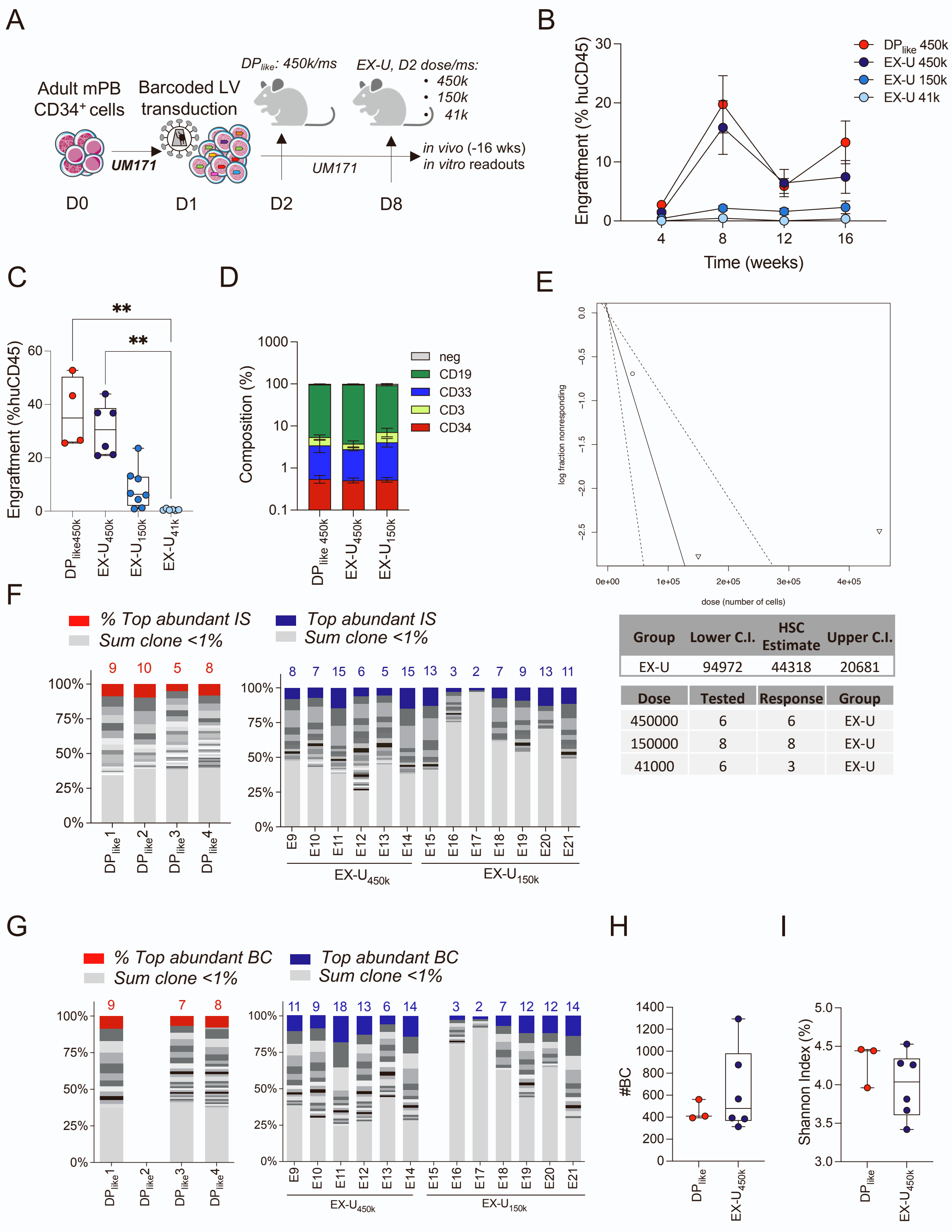

Group	Lower C.I.	HSC Estimate	Upper C.I.
EX-U	94972	44318	20681

Dose	Tested	Response	Group
450000	6	6	EX-U
150000	8	8	EX-U
41000	6	3	EX-U

Figure S1 - related to Fig.1. (**A**) Schematic representation of the experimental design. $CD34^+$ cells from the mPB of an adult healthy donor were transduced with a purified lentiviral vector expressing a highly diverse barcode and expanded in the presence of UM171 (EX-U) or not (DP_{like}). The indicated doses ($k = x10^3$) of DP_{like} and EX-U cells were xenotransplanted. (**B**) Percentage of human CD45+ cells (mean ± SEM) present in the peripheral blood (PB) of DP_{like} (n=4), EX-U_{450k} (n=6), EX-U_{150k} (n=8) and EX-U_{41k} (n=6) at the indicated number of weeks post-transplant. (**C**) Percentage of human CD45+ cells present in the bone marrow (BM) of DP_{like} (n=4), EX-U_{450k} (n=6), EX-U_{150k} (n=8) and EX-U_{41k} (n=6) at 16 weeks post-transplant (Kruskal-Wallis test with Dunn's multiple comparison). (**D**) Lineage composition (mean ± SEM) of the human $CD45^+$ cell graft in the BM at 16 weeks post-xenotransplantation. B cells: $CD19^+$; myeloid cells: $CD33^+$; T cells: $CD3^+$; $CD34^+$: HSPC cells; lineage negative: $CD19^-CD33^-CD3^-CD34^-$. (**E**) Top: Estimation of HSC frequency through Extreme Limiting Dilution Analysis (ELDA) transplantation dose response plots with estimated mean HSC frequency corrected on day 0 equivalent doses (full line) and 95% Wald confidence intervals (dashed lines) for tested samples. Engraftment was defined as ≥0.5% hCD45+ cells in mouse BM. Bottom: table of HSC estimates on day 0 equivalent doses with 95% Wald confidence intervals and dose-response table with number of tests and successful engraftment cases. (**F**) Relative abundance clonality analysis. Each bar represents the relative abundance of insertion sites from individual mice pertaining to the DP_{like} (left) or EX-U_{450K} and EX-U_{150K} groups (right). Bottom light grey stacks indicate the sum of insertions with <1% relative abundance, middle stacks indicate insertions with >1% abundance, where the top abundant insertions are highlighted in red or blue respectively. Number of retrieved top abundant insertions are reported above each column. (**G**) Same as in E, using the unique barcode as a clonal readout (Table S1). Samples MM2 and E15 did not pass prespecified quality thresholds and were discarded from downstream analysis. (**H**) Box plots indicating number of unique barcodes. Each dot represents

an individual mouse. (**I**) Box plots indicating the Shannon diversity index as measure of clonal population diversity analyzed by barcode. Each dot indicates an individual mouse of the indicated group.

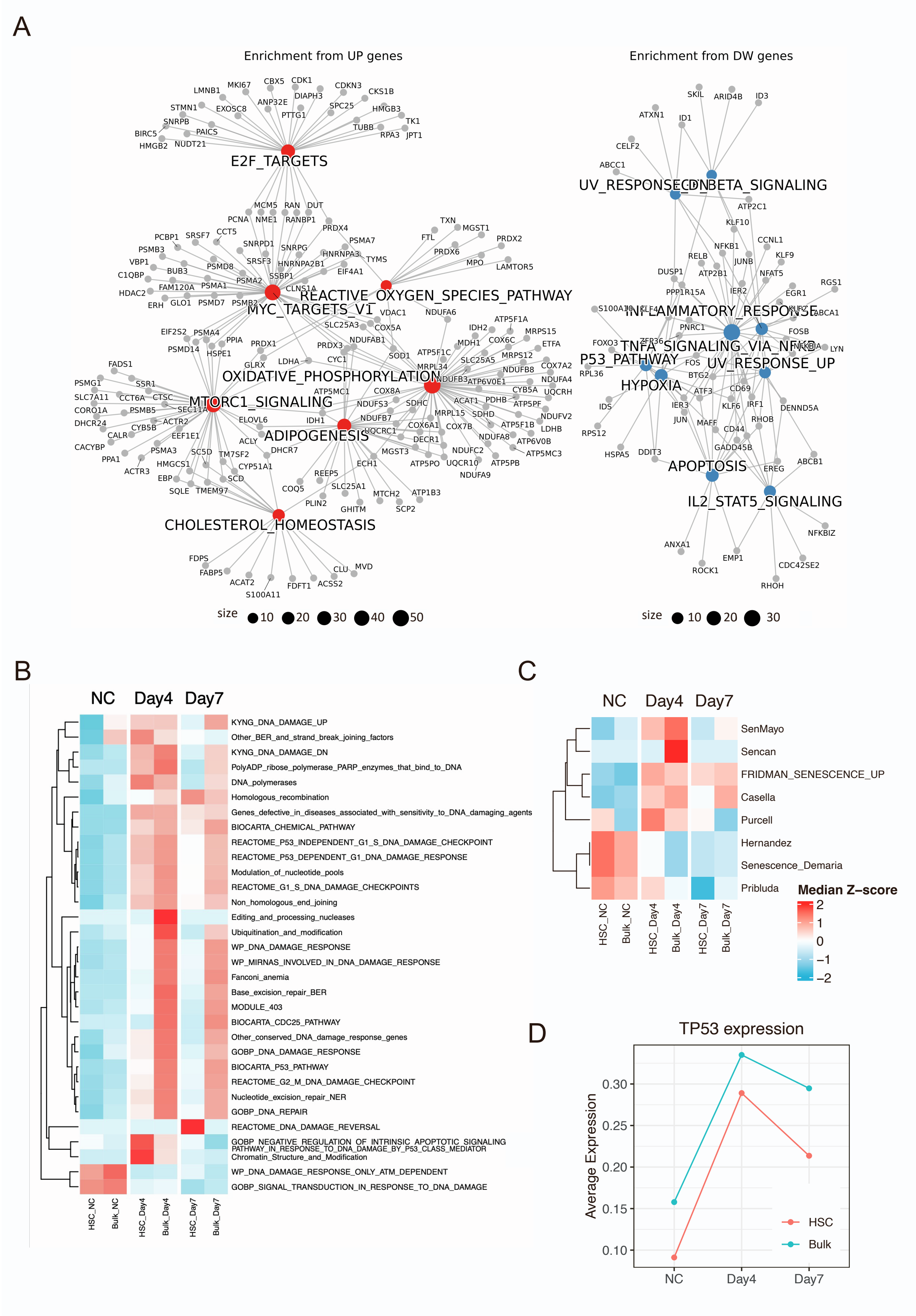
A
Enrichment from UP genes
Enrichment from DW genes
E2F_TARGETS
MYC_TARGETS_V1
REACTIVE_OXYGEN_SPECIES_PATHWAY
OXIDATIVE_PHOSPHORYLATION
MTORC1_SIGNALING
ADIPOGENESIS
CHOLESTEROL_HOMEOSTASIS
UV_RESPONSE_DN
TGF_BETA_SIGNALING
INFLAMMATORY_RESPONSE
TNFA_SIGNALING_VIA_NFKB
P53_PATHWAY
UV_RESPONSE_UP
HYPOXIA
APOPTOSIS
IL2_STAT5_SIGNALING
size 10 20 30 40 50
size 10 20 30
B
NC Day4 Day7
KYNG_DNA_DAMAGE_UP
Other_BER_and_strand_break_joining_factors
KYNG_DNA_DAMAGE_DN
PolyADP_ribose_polymerase_PARP_enzymes_that_bind_to_DNA
DNA_polymerases
Homologous_recombination
Genes_defective_in_diseases_associated_with_sensitivity_to_DNA_damaging_agents
BIOCARTA_CHEMICAL_PATHWAY
REACTOME_P53_INDEPENDENT_G1_S_DNA_DAMAGE_CHECKPOINT
REACTOME_P53_DEPENDENT_G1_DNA_DAMAGE_RESPONSE
Modulation_of_nucleotide_pools
REACTOME_G1_S_DNA_DAMAGE_CHECKPOINTS
Non_homologous_end_joining
Editing_and_processing_nucleases
Ubiquitination_and_modification
WP_DNA_DAMAGE_RESPONSE
WP_MIRNAS_INVOLVED_IN_DNA_DAMAGE_RESPONSE
Fanconi_anemia
Base_excision_repair_BER
MODULE_403
BIOCARTA_CDC25_PATHWAY
Other_conserved_DNA_damage_response_genes
GOBP_DNA_DAMAGE_RESPONSE
BIOCARTA_P53_PATHWAY
REACTOME_G2_M_DNA_DAMAGE_CHECKPOINT
Nucleotide_excision_repair_NER
GOBP_DNA_REPAIR
REACTOME_DNA_DAMAGE_REVERSAL
GOBP_NEGATIVE_REGULATION_OF_INTRINSIC_APOPTOTIC_SIGNALING_PATHWAY_IN_RESPONSE_TO_DNA_DAMAGE_BY_P53_CLASS_MEDIATOR
Chromatin_Structure_and_Modification
WP_DNA_DAMAGE_RESPONSE_ONLY_ATM_DEPENDENT
GOBP_SIGNAL_TRANSDUCTION_IN_RESPONSE_TO_DNA_DAMAGE
HSC_NC Bulk_NC HSC_Day4 Bulk_Day4 HSC_Day7 Bulk_Day7
C
NC Day4 Day7
SenMayo
Sencan
FRIDMAN_SENESCENCE_UP
Casella
Purcell
Hernandez
Senescence_Demaria
Pribluda
HSC_NC Bulk_NC HSC_Day4 Bulk_Day4 HSC_Day7 Bulk_Day7
Median Z-score
2
1
0
−1
−2
D
TP53 expression
Average Expression
0.30
0.25
0.20
0.15
0.10
NC Day4 Day7
HSC
Bulk

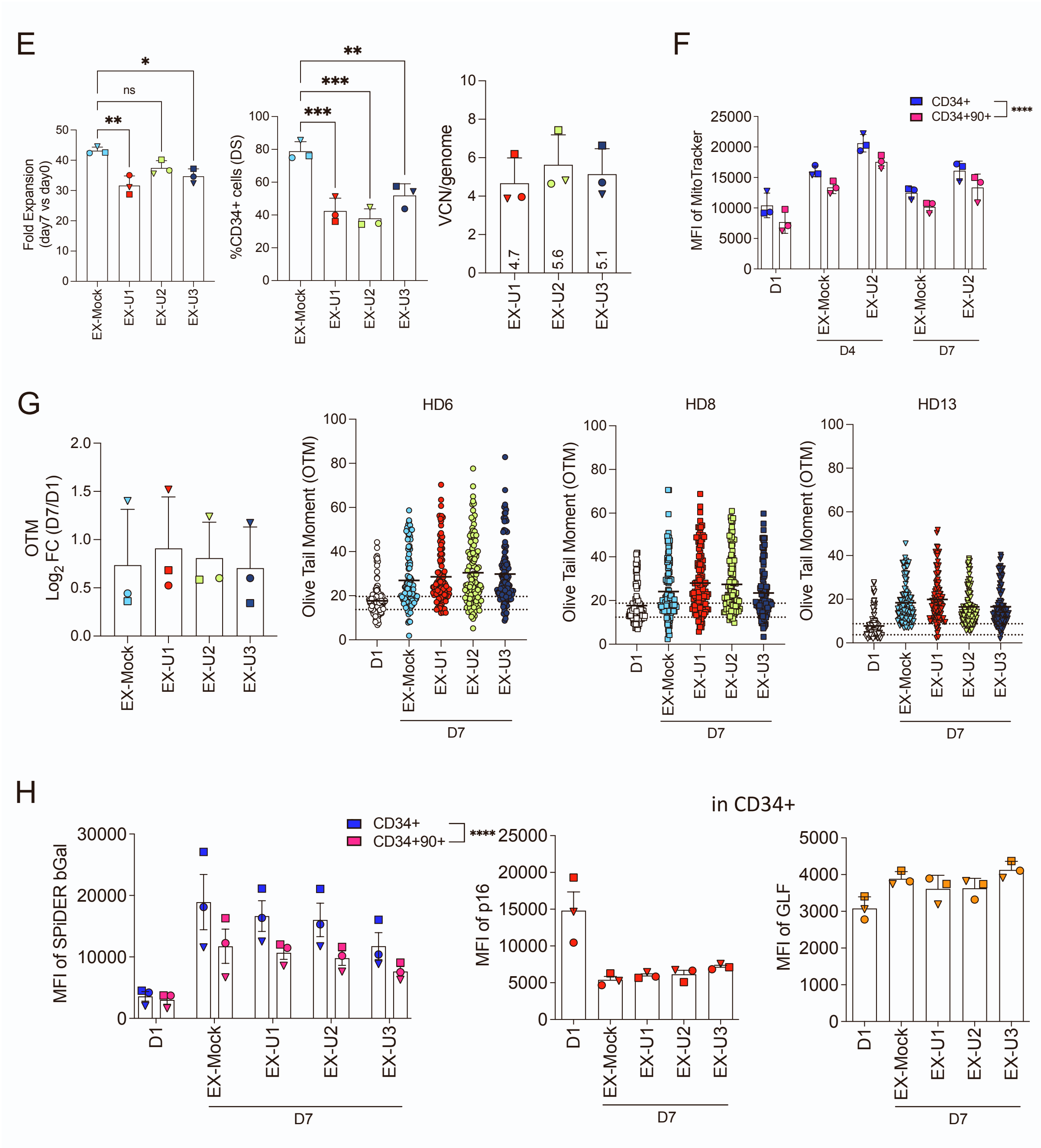

Figure S2 - related to Fig.2. (A) ORA with MSigDB hallmark pathways enriched from commonly up- or down-regulated genes over time (from uncultured to day 4 [early] or day 8 [late]) across populations. The cnetplots display enriched terms and their associated genes: upregulated genes are shown in the left plot, and downregulated genes in the right plot. Dot size indicates the number of genes associated with each term. For further details, see the scRNA-seq Methods section. (**B-C**) Heatmap of enrichment for DNA damage (B) and cellular

senescence (C) related gene signatures in HSC or bulk cell clusters either prior to ex-vivo expansion (NC: not cultured) or at different culture timepoints (D4 and D7) from the scRNAseq data described in Fig. 2C. Signatures were derived from curated reference gene lists (see Methods). (**D**) Mean single-transcript expression of *TP53* in HSC bulk (non-HSC) cell clusters prior to (NC) and at different timepoints of ex-vivo expansion (D4, D7) from the scRNAseq dataset of Fig. 2C. (**E**) In vitro expansion, immunophenotype, and transduction efficiency of mPB $CD34^+$ cells from n = 3 healthy donors cultured under EX-U conditions with lentiviral transduction at different prestimulation time windows (EX-U1: 24h, EX-U2: 36h or EX-U3: 72h) or mock (Mock). Left to right: fold expansion (vs day 0), percentage of $CD34^+$ cells at the end of culture, and vector copy number (VCN) per genome measured by ddPCR in transduced conditions. Each symbol represents one donor; bars indicate mean ± SEM. (**F**) Mitochondrial mass assessed by MitoTracker Green staining and flow cytometry in mPB $CD34^+$ cells from n=3 healthy donors at D1, D4, and D7 of EX-U culture, ±LV (EX-U2: 36h prestimulation). Geometric mean fluorescence intensity (gMFI) is shown for total $CD34^+$ cells and the $CD34^+CD90^+$ subset. Each symbol represents one donor. (**G**) DNA damage measured by alkaline COMET assay in mPB $CD34^+$ cells from n = 3 healthy donors. Left: intra-donor $\log_2$ fold change (D7/D1) of olive tail moment (OTM) across conditions. Right: single-cell OTM values at indicated time points (D1, D7) and culture conditions for each donor; each dot represents one analyzed cell. Bars indicate mean ± SEM where applicable. Dashed horizontal line represent the D1 25th and 75th percentile for each donor. (**H**) Flow cytometry–based quantification of senescence-associated markers in mPB $CD34^+$ cells from n=3 healthy donors at D1 and D7 of EX-U culture, ±LV at different prestimulation time windows (EX-U1: 24h, EX-U2: 36h or EX-U3: 72h). Left to right: senescence-associated β-galactosidase (SA-β-Gal) activity, intracellular p16, and lipofuscin (GLF) signal. SA-β-Gal is shown for both $CD34^+$ and $CD34^+CD90^+$ populations, including paired comparison between subsets; p16 and GLF are

shown for $CD34^+$ cells only. Each symbol represents one donor; bars indicate mean ± SEM. Statistical analysis, unless specified otherwise: paired Wilcoxon test.

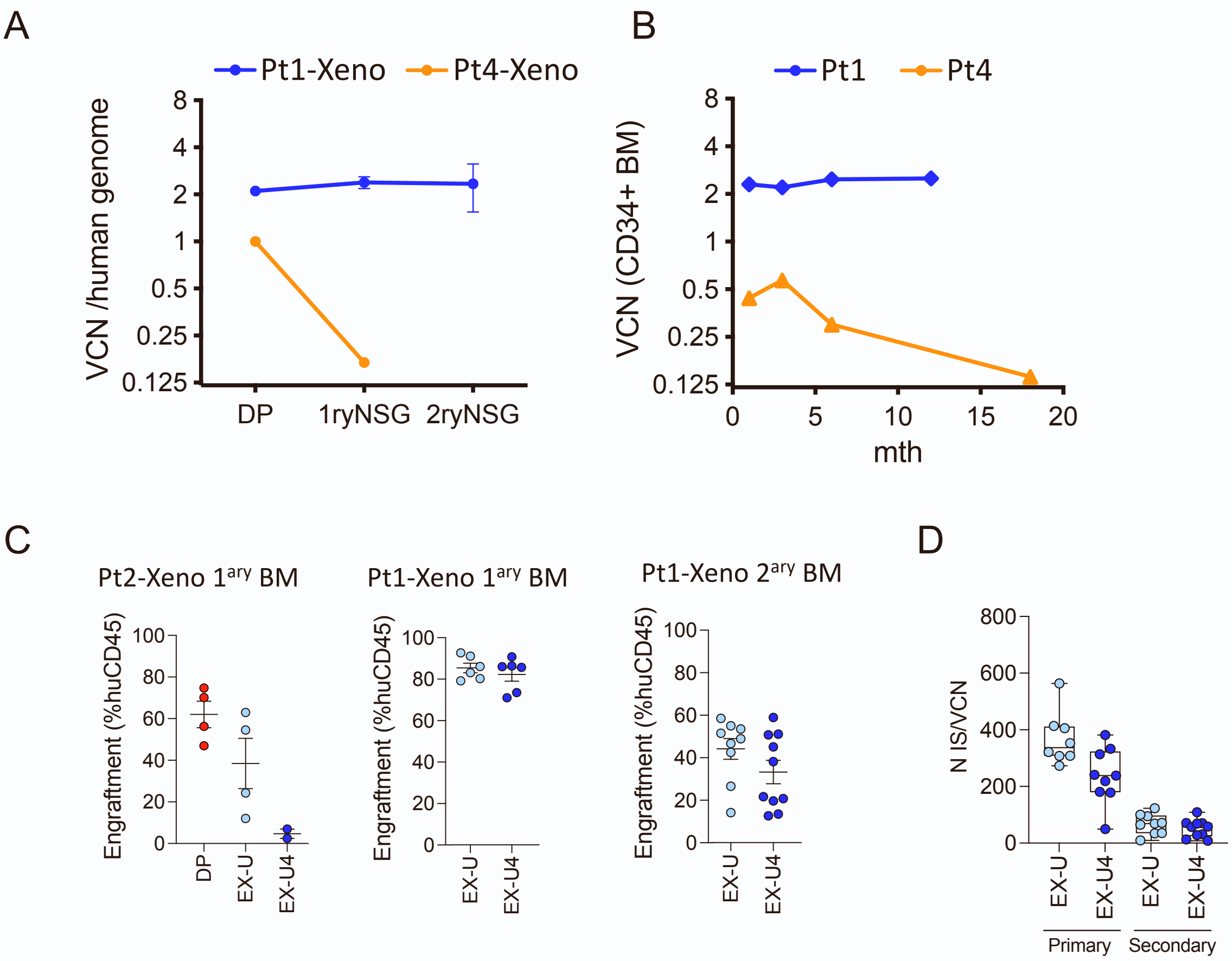

E

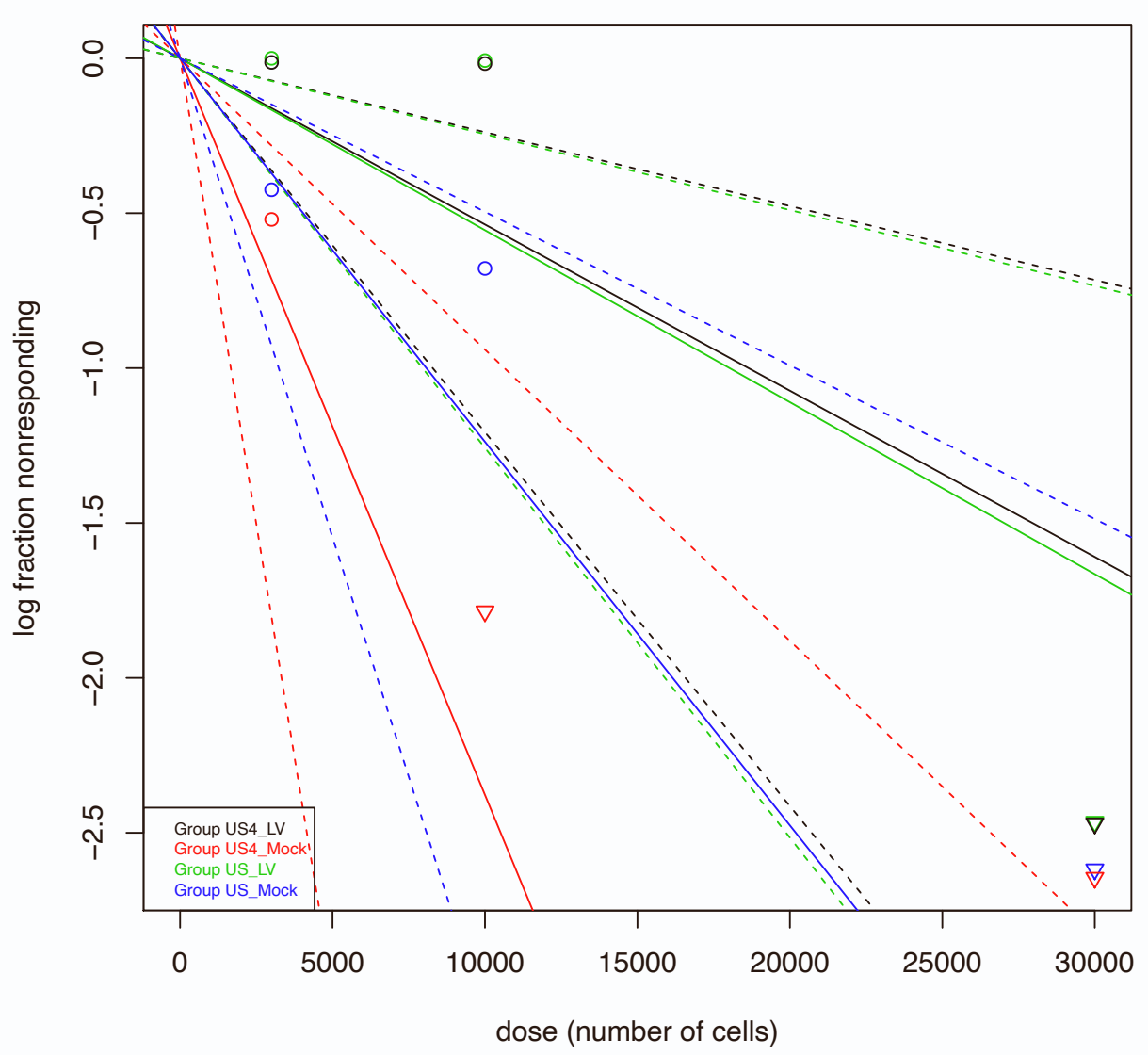

Dose	Tested	Response	Group
30000	7	7	US_Mock
10000	2	1	US_Mock
3000	3	1	US_Mock
30000	6	6	US_LV
10000	3	0	US_LV
3000	4	0	US_LV
30000	7	7	US4_Mock
10000	3	3	US4_Mock
3000	5	2	US4_Mock
30000	6	6	US4_LV
10000	3	0	US4_LV
3000	5	0	US4_LV

Group	Lower C.I.	HSC Estimate	Upper C.I.
US4_LV	41935	18640	8285
US4_Mock	10635	4206	1663
US_LV	40835	18018	7950
US_Mock	20174	8077	3234

Figure S3 - related to Fig.2 and Fig.4. (**A**) Transduction levels in the drug product (DP) (as measured by vector copy number analysis) for MPS patients Pt1 and Pt4 after 14-day in vitro culture or after xenotransplantation of the DP into primary NSG mouse recipients (1ryNSG; Pt1-Xeno: n=5 mice; Pt4-Xeno: n=8 mice) and after secondary transplantation (2ryNSG; Pt1-Xeno: n=3 mice) (mean ± SEM). (**B**) VCN in BM $CD34^+$ cells in the patients over time following gene therapy (subset of the data published in Gentner et al *(30)*, Figure S7). (**C**) Xenotransplantation of expanded DPs from MPS patients Pt1 and Pt2, expanded either in the presence of UM171 (EX-U, see Fig.1D, E) or a combination of UM171 and 4HPR (EX-U4). Human $CD45^+$ cell engraftment is shown in the bone marrow of primary (1^{ary}) recipients (Pt2-Xeno, 17 weeks; Pt1-Xeno, 12 weeks. Endpoint analysis) and secondary (2^{ary}) recipients of the human graft harvested from the bone marrow of 1^{ary} recipients from Pt1-Xeno (12 weeks endpoint). (**D**) Number of unique insertion sites normalized on vector copy number found in the BM of 1^{ary} and 2^{ary} recipient mice transplanted with the expanded DP from Pt1. See also Fig.1L). (**E**) Estimation of HSC frequency through Extreme Limiting Dilution Analysis (ELDA) transplantation dose response plots with estimated mean HSC frequency corrected on day 0 equivalent doses (full line) and 95% Wald confidence intervals (dashed lines) for tested samples. Engraftment was defined as ≥0.5% $hCD45^+$ cells in mouse BM. Bottom: table of HSC estimates on day 0 equivalent doses with 95% Wald confidence intervals and dose-response table with number of tests and successful engraftment cases.

SUPPLEMENTAL TABLES:

Table S1: LV Barcoding data shown in Fig S1F. Sheet 1 describes metadata associated with analyzed samples including group, VCN, % of engraftment and % of transduction (NGFR). This sheet is the sample sheet provided to the barcode data analysis script and includes the cutoff and MaxN variables representing the minimal % of abundance to retain a barcode and the number of maximum mice that can share a barcode within each group to be retained in the downstream analysis. Sheet 2 shows the raw data produced by barseq software in tabular format. Sheet 3 includes the filtered version of sheet 2 according to applied filters. Sheet 4 shows the complete processed data provided by the data analysis pipeline with all variables included. Sheet 5 shows the intragroup sharing percentage (1 mouse vs all intragroup). Sheet 6 summarizes the number of barcodes identified in each sample/group. Sheet 7 shows Shannon diversity indexes produced with vegan R package.

Table S2: scRNAseq data from Fig2C dataset. Sheet1 describes basic metrics including median number of UMIs and expressed genes per cell (nCount and nFeatures) as well as the % of mitochondrial genes per cell. Sheet 2 shows the cells distribution across samples and cell types, including the cell type % values within each sample used as input for generation of Fig.2C and Fig.2F. Sheet 3 includes cell type annotation according to cluster resolutions within the dataset metadata. Sheet 4 to 7 report marker genes according to different granularity of clustering and cell type annotations.

Table S3: scRNAseq data from Fig.2H dataset. describes basic metrics including median number of UMIs and expressed genes per cell (nCount and nFeatures) as well as the % of mitochondrial genes per cell. Sheet 2 shows the cells distribution across samples and cell types.

Sheet 3 and 4 show the distribution of WPRE positive and negative cells across timepoints and cell types for Pt1 and Pt4 respectively.

Table S4: LV Barcoding data from Fig. 3. Sheet 1 describes metadata associated with analyzed samples including group, VCN, % of engraftment and % of transduction (NGFR). This sheet is the sample sheet provided to the barcode data analysis script and includes the cutoff and MaxN variables representing the minimal % of abundance to retain a barcode and the number of maximum mice that can share a barcode within each group to be retained in the downstream analysis. Sheet 2 and Sheet 3 show the raw and filtered data output from barseq software. Sheet 4 shows the complete processed data provided by the data analysis pipeline with all variables included. Sheet 5 shows the intragroup sharing percentage (1 mouse vs all intragroup). Sheet 6 reports the number of barcodes identified in each sample/group. Sheet 7 shows the Shannon diversity indexes produced with vegan R package.

Table S5: Same as Table S4, comparing SCGM (EX-U) primary & secondary transplant samples and SCGM (EX-US) primary and secondary transplant samples.

Table S6: Same as Table S4, comparing transduction timing samples from DP-like-U1 to EX-U1, EX-U2 and EX-U3 groups.

Table S7: BulkRNA sequencing analysis comparing 72h vs 24h. Sheet 1 and 2 report differentially expressed genes in the two batches (1vs1). Sheet 3 and 4 report concordant up and down regulated genes from the 72h vs 24h comparison across the two different batches. Sheet 5 reports enriched terms from ORA using the reactome database. Sheet 6 shows the GSEA results from integrated batches using custom senescence reference signatures.

Table S8: For the cytokine optimization experiments (Fig. 3A–C), linear mixed-effects models were fitted using the lme4 and lmerTest R packages, with IL3 dose and IL6 dose as fixed effects (including their interaction) and donor as a random intercept. Overall effects were assessed by

Type III ANOVA with Satterthwaite's approximation for denominator degrees of freedom. Post hoc pairwise comparisons of estimated marginal means (emmeans package) were adjusted using the Holm method.
