# Supplementary material for: Ex vivo expansion of hematopoietic stem and progenitor cells from human mobilized peripheral blood for gene therapy applications
Source: Mol Ther. 2026 May 26;34(8):4581–603. doi: 10.1016/j.ymthe.2026.05.014 (PMC13464310; doi:10.1016/j.ymthe.2026.05.014)
Supplement: Supplementary file 2 [file mmc10.pdf]

Molecular Therapy
Original Article

Ex vivo expansion of hematopoietic stem and progenitor cells from human mobilized peripheral blood for gene therapy applications

Erika Zonari,[1,10] Matteo Maria Naldini,[1,2,10] Matteo Barcella,[1,10] Monica Volpin,[1,10] Francesca Vincenti,[1,7] Giacomo Desantis,[1] Leila Hadadi,[3] Carolina Caserta,[1] Ilaria Galasso,[1] Beatrice Martini,[1] Federico Midena,[1] Giacomo Farina,[1] Roberta Vacca,[1] Francesca Tucci,[1,4] Leonardo Ormoli,[1] Ilaria Visigalli,[1] Michela Vezzoli,[1] Dejan Lazarevic,[2] Ivan Merelli,[1,8] Stephanie Z. Xie,[5] John E. Dick,[5,6] Raffaella Di Micco,[1,9] Eugenio Montini,[1] and Bernhard Gentner[1,3]

[1]San Raffaele Telethon Institute for Gene Therapy (SR-TIGET), IRCCS San Raffaele Scientific Institute, 20132 Milan, Italy; [2]Vita-Salute San Raffaele University, 20132 Milan, Italy; [3]Ludwig Institute for Cancer Research and Department of Oncology, University of Lausanne (UNIL) and Lausanne University Hospital (CHUV), 1011 Lausanne, Switzerland; [4]Pediatric Immunohematology and Bone Marrow Transplantation Unit, IRCCS San Raffaele Scientific Institute, 20132 Milan, Italy; [5]Princess Margaret Cancer Centre, Toronto, ON M5G 2C4, Canada; [6]Department of Molecular Genetics, University of Toronto, Toronto, ON M5S 1A8, Canada; [7]University of Milan-Bicocca, Department of Informatics, Systems and Communication, 20126 Milan, Italy; [8]Institute for Biomedical Technologies (ITB) of the Italian National Research Council (CNR), 20054 Segrate, Italy; [9]University School of Advanced Studies IUSS, 27100 Pavia, Italy

***Ex vivo* expansion of mobilized peripheral blood (mPB) hematopoietic stem cells (HSCs) represents a promising approach to advance cell and gene therapy strategies yet is hampered by loss of stem cell function when applying commonly used culture protocols. We performed in-depth characterization of mPB expansion cultures by single-cell RNA sequencing, which highlighted differentiation trajectories with preservation of lineage fidelity in committed progenitors. Defining a putative HSC cluster allowed an estimation of transduction efficiency in *ex vivo* cultures, which correlated with long-term gene marking in xenografts and patients enrolled in a gene-therapy study. We then developed a clinically translatable, good manufacturing practice-compliant process to expand lentiviral (LV)-transduced HSCs from mPB of pediatric patients and adult donors by biologically informed protocol improvements of cytokine supplementation, media choice, timing of LV transduction, and combinations of small molecules preventing the activation of differentiation programs. Our optimized process outperforms validated state-of-the-art cord-blood expansion protocols when applied to mPB. LV integration site analysis and genomic barcode-based clonal tracking provided definitive proof for symmetric HSC self-renewal divisions occurring during *ex vivo* culture. These results warrant clinical testing of this HSC transduction/expansion process in an upcoming clinical gene-therapy trial for autosomal recessive osteopetrosis (EU CT 2024-518972-30).**

INTRODUCTION

Continuous production of blood and immune cells throughout an individual's life relies on a reserve of quiescent hematopoietic stem cells (HSCs) that may dynamically respond to hematopoietic demand by undergoing self-renewal or multilineage differentiation. This triple ability- quiescence, self-renewal and differentiation- is impressively recognizable after HSC transplantation, where a few thousand donor HSCs reconstruct and maintain a full blood and immune cell compartment in the recipient.[1,2] The functional potential of HSCs is intimately linked to a complex bone marrow (BM) niche.[3] Transplantation of unmodified or genetically engineered HSCs can be an effective treatment for hematological malignancies and non-malignant disorders[4] but may be limited by availability of human leukocyte antigen (HLA)-matched HSCs (in allogeneic settings) or yield of genetically engineered cell products (in autologous gene therapy settings). *Ex vivo* expansion of HSCs aims to overcome such limitations by increasing numbers of functional HSCs/progenitors and/or enhancing their engraftment potential, thus potentially widening access to HSC-based treatments.

One clinically relevant application for *ex vivo* expansion is allogeneic umbilical cord-blood (CB) transplantation, where the small size of most CB units precludes their safe clinical use, especially in adults. This bottleneck could be overcome by *ex vivo* expansion before infusion into patients, allowing for a potentially broader choice of CB units with improved HLA matching.[5–8] Successful approaches have leveraged cytokine stimulation to promote CB HSC proliferation

Received 10 October 2025; accepted 22 May 2026;
https://doi.org/10.1016/j.ymthe.2026.05.014.

[10]These authors contributed equally

Correspondence: Bernhard Gentner, San Raffaele Telethon Institute for Gene Therapy (SR-TIGET), IRCCS San Raffaele Scientific Institute, 20132 Milan, Italy.
E-mail: bernhard.gentner@chuv.ch

© 2026 The Authors. Published by Elsevier Inc. on behalf of The American Society of Gene and Cell Therapy.
This is an open access article under the CC BY license (http://creativecommons.org/licenses/by/4.0/).

and survival, combined with molecules that counteract HSC commitment to differentiation, the default program triggered under these culture conditions, during which protein synthesis, signaling, and stress responses are upregulated.[9–11] Molecules shown to help maintain CB HSCs modulate histone modifiers,[12–16] the aryl hydrocarbon receptor pathway,[17,18] metabolism and organelle biology,[19–21] and Notch signaling.[5,22] Many of these strategies have shown functional evidence for some degree of HSC expansion when using CB as a source. Still, it remains largely unclear whether the combination of molecules acting through different mechanisms could further improve HSC expansion and whether these protocols perform similarly on other HSC sources. Another approach has focused on chemically defined culture media titrating the activation of essential signaling pathways and minimizing batch variability and contaminants often associated with products of biological origin.[23] Experimental research approaches are also exploring more complex systems, such as 3D cultures in hydrogels[24] or co-culture with HSC-supporting scaffolds or stromal cells mimicking the BM niche.[25,26] While promising, these models present significant limitations in terms of translational potential toward clinical applications, which require scalability and compliance with good manufacturing practice standards.

Another emerging application for *ex vivo* expansion is gene therapy (GT) of the patient's own HSCs, which is associated with less morbidity and potentially higher efficacy compared to allogeneic transplantation, when it comes to the treatment of select monogenic disorders.[27] In GT protocols, HSCs are genetically modified by vector mediated addition of a therapeutic gene or edited for correction of genetic defects.[28] However, GT protocols may reach suboptimal gene-transfer/correction efficiencies or may involve manipulation of autologous patient HSCs with low yield (e.g., in very young pediatric patients ineligible for leukapheresis or individuals with poor stem cell mobilization potential) or disorders where HSC quantity and quality are inherently compromised, such as reversible niche defects, BM failure syndromes, or chronic inflammatory conditions. *Ex vivo* expansion applied to GT protocols may allow to expand and/or select for genetically modified HSCs to achieve sufficient transgene-carrying cells for therapeutic efficacy. Furthermore, expansion of corrected HSCs may allow downscaling of transduction and editing steps reducing manufacturing costs. Hence, there are multiple arguments for applying *ex vivo* HSC expansion protocols in the autologous HSC gene-therapy context. Unfortunately, autologous CB units are generally not available as an HSC source. Mobilized peripheral blood (mPB)-derived HSCs are currently the preferred cell source for most clinical gene-therapy applications due to their easy access and their proven robust engraftment, faster neutrophil and platelet recovery, increased clonal diversity, and better transduction as compared to BM-derived HSCs.[29,30] Although a previous study has examined whether HSC expansion protocols developed for CB may sustain stemness in mPB HSCs,[31] functional evidence for *ex vivo* expansion of mPB or BM HSCs remains limited, and the underlying biological differences of such sources, including different proliferative capacity, differentiation priming, and progenitor composition, may impact the response to *ex vivo* stimulation.[32] Likewise, the impact of the genetic-engineering step on *ex vivo* expansion protocols is poorly characterized.

With the objective to optimize an *ex vivo* expansion protocol for lentiviral (LV)-transduced mPB HSCs for clinical use, we performed an in-depth characterization of LV-transduced, *ex vivo*-expanded mPB-HSCs from pediatric patients enrolled in a gene-therapy trial (NCT03488394) and from adult healthy donors, including single-cell RNA sequencing (scRNA-seq) and clonal graft analysis in primary and secondary xenografts.

While showing robust evidence for symmetric HSC division in *ex vivo* culture, we found that net HSC expansion is considerably lower compared to the data described for CB. To improve net mPB-HSC expansion, we explored combinations of HSC-maintaining molecules and undertook a hypothesis-driven, iterative optimization of the culture protocol toward the development of a clinically applicable process to produce LV-transduced, *ex vivo*-expanded HSCs from mPB.

RESULTS

UM171 *ex vivo* expansion results in net mPB-derived HSC maintenance from healthy donors and gene-therapy patients

To test previously described *ex vivo* expansion protocols, developed for umbilical CB[12] on mPB-derived HSCs, a clinically relevant HSC source in a gene-therapy context, we cultured hematopoietic stem and progenitor cells (HSPCs) from n = 5 mPB drug products (DPs) from a pediatric gene-therapy trial (NCT03488394) for mucopolysaccharidosis type I (MPS)[30] for 6–7 days using commercial media supplemented with early acting cytokines (SFT6: stem cell factor (SCF), 100 ng/mL; Fms-related tyrosine kinase 3 ligand (FLT3L), 100 ng/mL; thrombopoietin (TPO), 50 ng/mL; interleukin [IL]-6, 50 ng/mL), with or without the epigenetic modifier UM171 (Figure 1A). DP-derived cells expanded 8- to 27-fold (Figure 1B) and, as expected, the addition of UM171 increased the proportion of cells with a primitive immunophenotype compared to cytokine-only cultures (Figure 1C).

To assess the repopulating potential of MPSI-H patient-derived DPs expanded in the presence of UM171 (EX-U), we measured human cell engraftment and lineage composition at 12 weeks after xenotransplantation into primary (readout for short term (ST)-HSC; Figures 1D–1F) and secondary recipient immunodeficient mice (readout for long-term (LT) HSC; Figures 1E and 1G). EX-U cells were compared, at matched pre-expansion cell doses ("day-0 equivalent"), to their respective non-expanded DPs and the non-cultured (NC) CD34$^+$ cells used as starting material for the *ex vivo* engineering/culture process. All conditions resulted in detectable human cell engraftment in the BM of primary and secondary recipients (Figures 1D and 1E), with multilineage output (Figures 1F and 1G). *Ex vivo* culture had a different impact on ST-HSC function from the DPs of the two patients, resulting in a similar-sized primary graft for the DP from patient 1 (Pt1) (Figure 1D, left) but

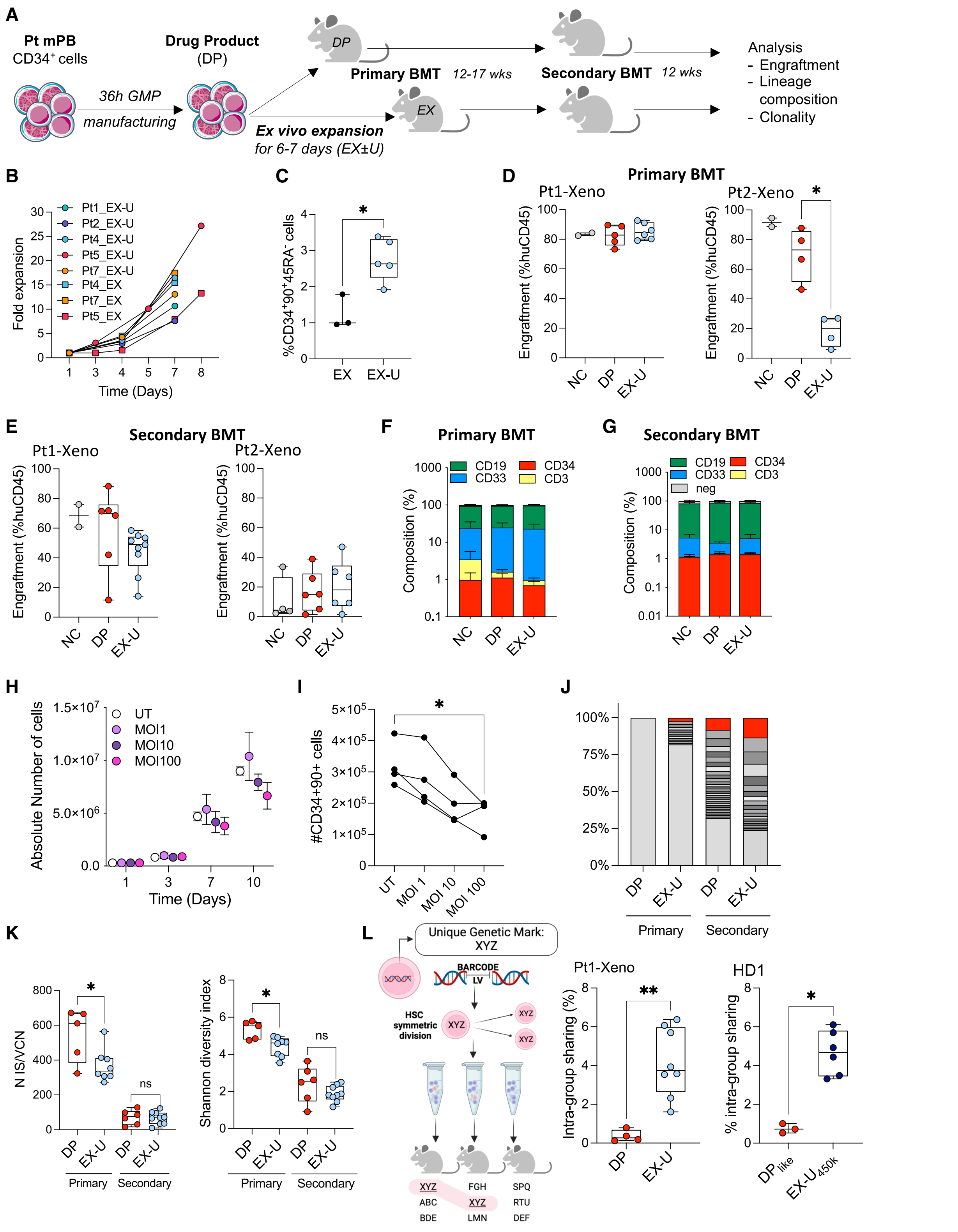

(legend on next page)

significantly reduced engraftment for the DP from Pt2 (Figure 1D, right). Nevertheless, proficient transfer of the grafts from the two patients to secondary recipients (Figures 1E and 1G) suggested that *ex vivo*-expanded cells maintained LT-HSC potential.

We hypothesized that the reduced primary engraftment of EX-U cells from Pt2 in xenotransplantation assays could be related to LV transduction when coupled to *ex vivo* expansion, as this patient had the most highly transduced DP in the study, with an average Vector Copy Number (VCN) per cell of 5.2, compared to a median VCN of 2.2 when considering all the eight treated patients.[30] To further test this hypothesis, we transduced mPB CD34$^+$ cells from four healthy adult donors with increasing LV doses observing, similarly to the DP of Pt2, a dose-dependent decrease in cell growth during *ex vivo* culture (Figure 1H), with concomitant reduction in the absolute number of immunophenotypically more primitive cells (Figure 1I). Thus, *ex vivo* culture may amplify well-described HSPC stress responses induced by genetic-engineering technologies particularly impacting ST-HSC function,[33] investigated in more detail below.

Next, we analyzed LV vector integration sites (LVISs) to determine the clonal structure of EX-U versus DP-derived grafts from Pt1. LVISs distribute semi-randomly across the genome. Hence, each individual transduced clone is characterized by one or more unique integration site(s) stably transmitted to the progeny. Xenografts derived from DP and EX-U had a highly polyclonal pattern in the primary mice, without detection of dominant clones (Figure 1J). As expected from a procedure enriching for LT-HSCs, secondary grafts had a less polyclonal composition in both groups but still showed no signs of clonal dominance (Figure 1J). Quantitative comparison between EX-U and DP showed that primary, but not secondary, grafts originating from EX-U had significantly fewer unique integration sites and a lower Shannon diversity index, suggesting some loss of ST-HSC activity also for cells from Pt1 during *ex vivo* expansion. Comparison of secondary recipients, a stringent approach for assessing LT-HSC output, showed preserved LT engraftment potential (Figure 1K).

To confirm the broader applicability of our expansion protocol to adult mPB, we compared the engraftment potential of EX-U and DP_{like} HSPCs from an adult healthy donor (HD1) after transduction with a highly diverse LV barcode library, simulating a gene-therapy context and allowing simultaneous clonal graft analysis by an independent methodology (Figure S1A). EX-U were xenotransplanted at decreasing day-0-equivalent cell doses, resulting in a dose-dependent decrease in peripheral blood human cell engraftment (Figure S1B). After 16 weeks from xenotransplantation, EX-U showed comparable engraftment to DP_{like} cells when injecting the same day-0 dose equivalent (Figure S1C), yet lower but still detectable multilineage engraftment (Figure S1D) when decreasing the dose to 33% and 9% of the DP_{like} input dose. Extreme limiting dilution analysis (ELDA) estimated an HSC frequency of 1 in 44,318 when calculated on the day-0-equivalent dose (Figure S1E). We assessed graft clonality by two methods, either by LVIS or by quantifying the abundance of individual barcodes introduced into the cells by the LV vector barcode (LVBC) library. Both methods consistently showed polyclonal reconstitution even at the intermediate expanded cell dose, with a concordant and strikingly similar clonal abundance pattern supporting the robustness of the two methods (Figures S1F and S1G; Table S1). Clonal diversity and the estimated number of human repopulating clones per mouse were equivalent or higher

Figure 1. Net HSC maintenance in UM171-expanded mPB CD34$^+$ cells

(A) Schematic representation of the experimental design. (B) *Ex vivo* expansion of drug products (DPs) from different MPS1 patients (Pt) for 6–7 additional days in the presence (dot; EX-U) or absence (square; EX) of UM171 (35 nM). Fold expansion is calculated as a ratio between final cell count on day7/8 with respect to seeding on day 1. (C) Fraction of HSC-enriched CD34$^+$CD90$^+$CD45RA$^-$ cells at the end of expansion culture of the samples from (B). *$p < 0.05$, Mann-Whitney test. (D) Human CD45$^+$ cell engraftment in the BM of NSG mice at 12 weeks post intravenous (i.v.) injection of the following mPB-derived HSPC products from 2 MPS1 patients: Pt1-Xeno (1) 400,000 non-cultured CD34$^+$ cells (NC; $n = 2$), (2) 400,000 DP cells (DP; $n = 5$), or (3) 4,000,000 *ex vivo*-expanded cells (outgrowth of 400,000 DP cells; EX-U; $n = 6$). Pt2-Xeno (1) 150,000 non-cultured CD34$^+$ cells (NC; $n = 2$), (2) 150,000 DP cells (DP; $n = 4$), or (3) 1,100,000 *ex vivo*-expanded cells (outgrowth of 150,000 DP cells; EX-U; $n = 4$). *$p < 0.05$, Mann-Whitney test. BM cells were obtained as terminal procedure or BM aspirate for the mice injected with cells from Pt1 and Pt2, respectively. See Figure S3 for the endpoint analysis of Pt2-Xeno. (E) Secondary transplantation of 2 and 1 × 10^6 CD34$^+$ cells isolated from primary NSG recipients engrafted with HSPC from Pt1-Xeno and Pt2-Xeno, respectively. BM engraftment of secondary recipients was assessed at 12 weeks post transplantation, respectively. Each dot represents an individual mouse. (F and G) Lineage composition of the human CD45$^+$ cell graft from (D) and (E), respectively. CD19$^+$, B cells; CD33$^+$, myeloid cells; CD3$^+$, T cells; CD34$^+$, HSPC; neg, CD19$^-$CD33$^-$CD3$^-$CD34$^-$ cells. Shown is the mean ± SEM from the pooled individual mice from Pt1-Xeno and Pt2-Xeno. (H) *Ex vivo* expansion (mean ± SEM) of mPB CD34$^+$ cells from $n = 4$ adult donors under EX-U conditions following transduction with a LV vector at increasing multiplicities of infection (MOI). UT, non-transduced cells. (I) Absolute number of CD34$^+$ CD90$^+$ cells on day 7 of expansion culture from (H). Each line represents a donor (*$p < 0.05$, Kruskal-Wallis test with Dunn's multiple comparison). (J) Relative abundance of unique LV integration sites (LVIS) from DP and EX-U groups from Pt1-Xeno in primary and secondary recipient mice. Bottom light gray stacks indicate the sum of integrations with <1% relative abundance, middle stacks indicate insertions >1% abundance, whereas the top abundant integrations are highlighted in red. (K) Left: box plots indicating number of integration sites normalized on transduction efficiency (VCN). Right: box plots indicating the Shannon diversity index as measure of clonal population diversity. Each dot represents an individual mouse of the treatment group: DP and EX-U in primary and secondary recipient, respectively (*$p < 0.05$, Mann-Whitney test). (L) Left: schematic representation on how unique LVIS or LV vector barcodes (LVBC), introduced at transduction, can be exploited to prove symmetric HSC divisions in culture by retrieving, at the experiment endpoint, the same genetic mark in two different mouse recipients transplanted with cells from a given *ex vivo* culture batch. Right: boxplots showing the proportion of shared LVIS (Pt1-Xeno) or shared LVBCs (HD1) between at least two individual mice receiving the same day-0-equivalent CD34$^+$ cell doses from the indicated HSPC products (intragroup sharing). Each dot represents the percentage of LVIS/LVBCs shared with at least one other mouse in the same experimental group. (*$p < 0.05$, **$p < 0.01$, Mann-Whitney test). Further details on HD1 are presented in Figure S1. In all box plots shown in the figure, the whiskers represent the minimum and maximum values of the data.

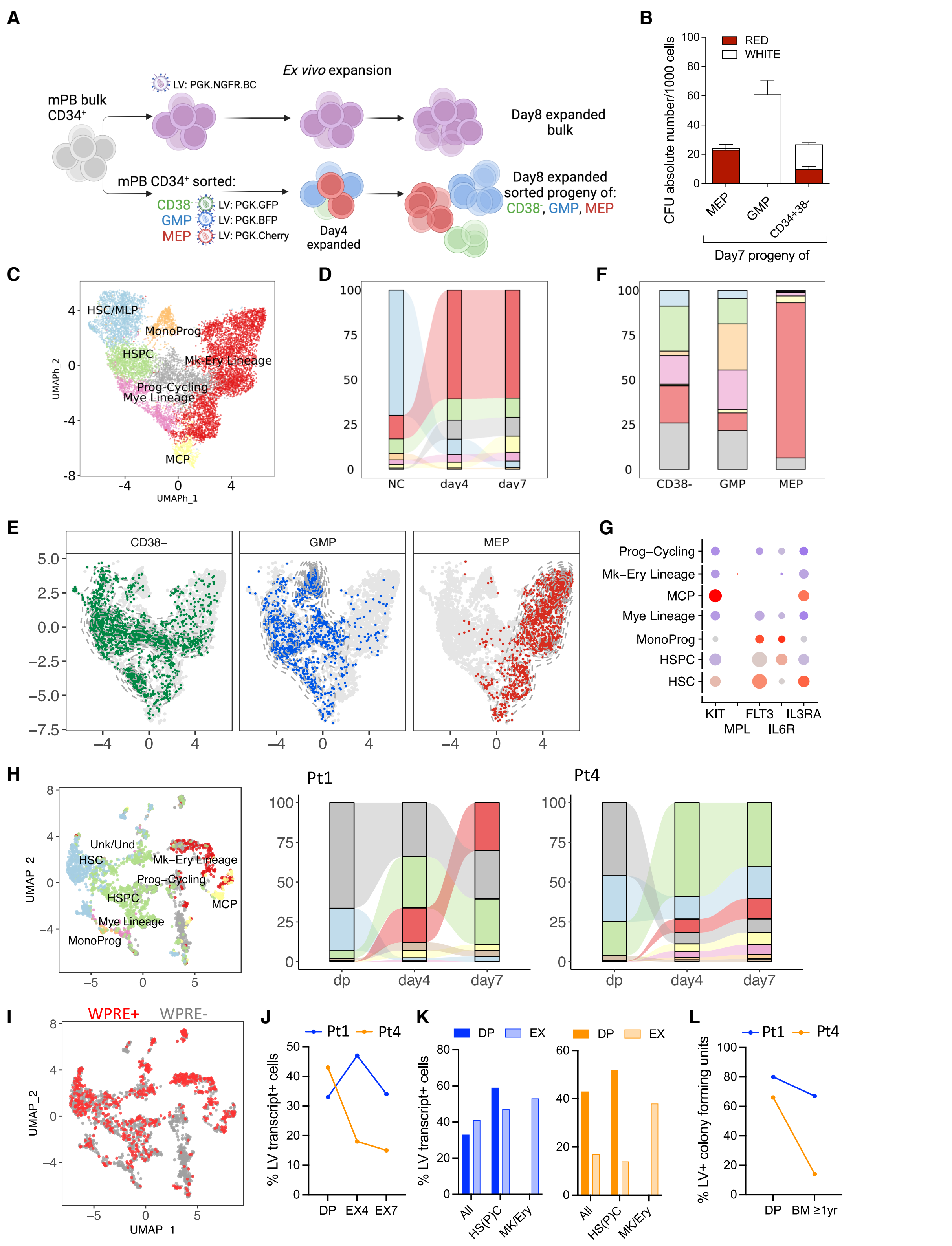

(legend on next page)

in the EX-U compared to the DP_{like} group at equivalent day-0 starting cell doses in line with at least LT-HSC maintenance during *ex vivo* culture (Figures S1H and S1I). To prove that some of these HSCs had undergone symmetric self-renewal divisions during *ex vivo* expansion, we identified unique LVISs or LVBCs from Pt1 and HD1, respectively, that could be detected in different mice injected with the same cell-therapy product (Figure 1L). Only background levels of such shared clones were detected in mice transplanted with DP (DP_{like}) cells, where cell counts remained stable during the short duration of culture, while 2%–7% of clonal markers were shared between mice that received EX-U cells (Figure 1L; Table S1).

Overall, these data suggest that the UM171 culture allowed net maintenance of adult and pediatric HSC from mPB, likely due to some HSCs undergoing symmetric division *ex vivo* and others being lost during the culture process. *Ex vivo* culture did not result in clonal dominance, preserving clonal diversity.

Ex vivo culture of mPB CD34$^+$ cells mostly expands committed progenitors with preserved lineage fate and allows prediction of *in vivo* DP characteristics by scRNA-seq

To characterize mPB CD34$^+$ cell population dynamics during EX-U culture, we performed scRNA-seq on cells from two adult healthy donors. CD34$^+$ cells from donor 1 were analyzed before culture, on day 4, and on day 8 of expansion. For donor 2, in addition to the CD34$^+$ bulk expansion culture, part of the CD34$^+$ cells were sorted by fluorescence-activated cell sorting (FACS) into subpopulations enriched in multipotent HSPC (CD34$^+$CD38$^-$), committed granulocyte-monocyte progenitors (GMPs) or megakaryocyte-erythrocyte progenitors (MEPs), and each subpopulation was marked by transduction with an LV expressing a different fluorescent protein for tracing back the population of origin prior to remixing the subpopulations together for expansion (Figure 2A). Colony-forming cell assay confirmed functional enrichment of, respectively, GMPs and MEPs post sorting (Figure 2B). A single object containing single-cell transcriptomes from all samples was generated following batch correction and dimensionality reduction. Unsupervised clustering was performed, and cellular states were manually annotated (Figure 2C; Table S2). Compared to NC cells, presumably more enriched in primitive cells, HSCs and multi-lymphoid progenitors (MLPs) progressively diminished over time in culture, while progenitor cell states, erythroid/megakaryocytic, actively cycling myeloid progenitors, and mast cell precursors (MCPs) increased (Figure 2D). These data suggest that expansion culture mainly supported the growth of mPB-derived progenitors, rather than more primitive HSCs, which are progressively diluted during culture. We next asked whether committed progenitors were able to maintain lineage fate during culture or whether there was plasticity. The expansion culture output of day-0-sorted GMPs and MEPs faithfully mapped on the myeloid/monocytic and erythroid/megakaryocyte progenitor clusters, respectively (Figures 2E and 2F). The day-0 CD34$^+$CD38$^-$ progeny lacked the most differentiated states, showed a bias for the myeloid progenitor area, and maintained a sizable fraction of cells within the HSC cluster (Figure 2F), in line with our previous data advocating the CD34$^+$CD38$^-$ population as an HSC-enriched starting population for expansion cultures.[31] Taken together, these data support that HSPC fate is maintained during expansion culture.

To evaluate the overall impact of *ex vivo* culture on HSPC transcriptomes, we identified longitudinally deregulated genes common across HSPC populations in the scRNA-seq dataset and assessed for enrichment of hallmark gene expression signatures (Figure S2A; Table S2). Hallmarks enriched from upregulated genes over time included those related to proliferation, oxidative metabolism,

Figure 2. Characterization of expansion cultures by scRNA-seq

(A) Adult mPB CD34$^+$ cells from n = 2 healthy donors were expanded with UM171 in bulk following transduction with a barcoded LV library (upper panel) or after marking FACS-sorted GMPs (CD34$^+$CD38$^+$CD45RA$^+$ or CD34$^+$CD38$^+$CD45RA$^-$BAH1$^-$CD71$^-$), MEPs (CD34$^+$CD38$^+$CD45RA$^-$CD71$^+$), or multipotent HSPCs (CD34$^+$CD38$^-$) with a color-coded LV expressing a blue (BFP), red (Cherry), or green (GFP) fluorescent protein, respectively (lower panel). Cells were harvested on day 4 and/or day 8 for scRNA-seq library preparation. (B) Colony-forming cell assay on sorted progeny of GMPs, MEPs, and CD38$^-$ cells after 7 days of expansion. Absolute number of colony-forming units (CFU) with red (BFU-E) or white (CFU-G/M) morphology are shown for the three populations of origin (mean ± SEM, n = 5 technical replicates). (C) Uniform manifold approximation and projection (UMAP) encompassing the samples described in (A) with manually curated cell type annotation, as described in materials and methods. HSC/MLP, hematopoietic stem cells/multi-lymphoid progenitors; HSPC, hematopoietic stem/progenitor cells; Mye, myeloid lineage progenitors; Mono, monocyte progenitors; MCP, mast cell progenitors; Mk-Ery, megakaryocyte/erythroid lineage progenitors; Prog-Cycling, progenitors predicted to be in active phases of the cell cycle. (D) Alluvial plot showing population frequencies over time, calculated within each time point. NC, non-cultured sample (day 0). (E) Distribution of the expanded day-7 progeny of FACS-sorted cell populations across UMAP embedding according to cell population of origin and (F) relative frequency of scRNA-seq-based cell type annotations as defined in (C). (G) Expression of key cytokine receptors in the indicated cell type annotations. The color palette (from blue to red) represents the Z score of receptor expression (from low to high, respectively), while the size of the dots represents the percentage of cells expressing the indicated cytokine receptor transcript in the indicated population. (H) Pediatric mPB DPs from n = 2 MPS1 patients (Pt1, Pt4) were sorted for CD34$^+$CD90$^+$CD45RA$^-$ cells (dp = day 1) or expanded for 4 (EX4) and 7 days (EX7) in UM171 before sorting into the CD34$^+$CD90$^+$CD45RA$^-$ cell fractions. scRNA-seq was performed on the HSC-enriched subpopulations at the three time points. The UMAP on the left shows automated cell type annotation using scGate leveraging the adult mPB dataset described in (A)–(G) as reference (see materials and methods for details). The alluvial plots on the right show time-dependent changes in population frequencies. Note that CD34$^+$CD90$^+$CD45RA$^-$ cells, an HSC-enriched subset of the cultures, were analyzed here. (I) Cells with LV-derived transcripts (*WPRE*$^+$) are projected on the UMAP plot as a surrogate for estimating transduction efficiency (TE). (J) Proportion of LV transcript+ cells over time in the two patients. TE over time and across major populations. (K) Proportion of LV transcript+ cells in the DP (intensely colored bar) vs. the expanded cells (faintly colored bars; EX4 and EX7 samples were aggregated) for the entire population (All) or within the indicated, scRNA-seq-defined subpopulations. HSCs and HSPCs were aggregated to obtain a representative number of cells for all conditions. Left graph (blue bars), Pt1; right graph, orange, Pt4. (L) TE assessed by digital droplet PCR on individually plucked colony-forming units (gold-standard assay), either on the DP (bulk) or in the BM of the patients at 1 year after gene therapy, as reported in Gentner et al.[30]

mTORC1 signaling, and lipid metabolism, while hallmarks associated with inflammation and immediate-early response genes were suppressed. DNA damage- and senescence-associated gene signatures were also enriched during culture, with a noticeably stronger induction in the bulk HSPC population compared to the molecularly defined HSC cluster, a peak at day 4 concomitant with highest TP53 levels, and partial resolution by day 7 (Figures S2B–S2D). To confirm these transcriptomic data, we performed MitoTracker staining, alkaline COMET assays, and measured protein markers of senescence on mPB CD34$^+$ cells (n = 3 donors) during culture, with or without LV transduction (Figures S2E–S2H). Mitochondrial mass increased, peaking on day 4, more strongly in CD34$^+$ bulk cells than in the primitive compartment and further boosted by LV transduction (Figure S2F). Culture was also associated with increased olive tail moment, with a trend toward higher values upon LV transduction (Figure S2G). Increased SA-β-galactosidase activity with moderate increase in lipofuscin, but a decrease of p16INK4a in the expanded population, argued in favor of a transient stress response rather than irreversible senescence, independently from LV transduction (Figure S2H).

To evaluate the influence of cytokine supplementation on culture dynamics, we assessed the expression of key cytokine receptors on different cell populations in scRNA-seq. In addition to KIT and FLT3, IL-3RA was also highly expressed in the most primitive annotated HSC subpopulation, while the IL-6 receptor was predominantly detected in the HSPC and monocyte progenitors (Figure 2G). These findings prompt additional investigations on the role of IL-6 and IL-3 supplementation during mPB expansion (see below).

Next, we hypothesized that in-depth characterization of expansion cultures by single-cell transcriptomics could aid in understanding DP characteristics where current standard release assays may not fully correlate with *in vivo* behavior. Although HSC-GT treatment in the MPSI-H trial resulted in encouraging metabolic correction and early clinical outcomes in all treated patients as of an interim analysis, the *in vivo* VCN stabilized in three out of eight patients at values below 0.3, while *in vitro* assays on the DPs measured a transduction efficiency >60% and VCN $\geq$ 1.[30] Xenotransplantation accurately recapitulated steady-state VCN in patients (Figure S3A), thus confirming that the VCN drop upon transplantation in the three patients was not due to reduced conditioning. We performed scRNA-seq on CD34$^+$CD90$^+$CD45RA$^-$, HSC-enriched populations from DP, day-4 EX-U, and day-7 EX-U cultures from two representative MPS patients, namely Pt1 (correspondence between *in vitro/in vivo* VCN) and Pt4 (VCN drop upon transplantation). Cell populations were annotated by label transfer from the dataset of Figure 2C (Figure 2H; Table S3). The DPs from the two patients had similar compositions, and EX-U showed the expected population dynamics, with a contraction of the HSC population (more in Pt1 than in Pt4) and an expansion of HSPC and Ery/Mk progenitors (more in Pt1). By assessing expression of LV-specific transcripts (*WPRE, Woodchuck Hepatitis Virus Posttranscriptional Regulatory Element*) at single-cell resolution (Figure 2I), we were able to estimate the percentage of LV$^+$ transcriptomes over time and within specific cell clusters as a putative surrogate for transduction efficacy (Figure 2J; Table S3). Strikingly, we recapitulated a drop in WPRE$^+$ cells during EX-U for Pt4, but not Pt1, and this was evident in HS(P)C but not Ery/MK progenitors (Figure 2K; Table S3), resembling the drop in LV$^+$ colony-forming units (CFU) in the BM of the patient with respect to the DP (Figure 2L). These data highlight the potential of scRNA-seq of expansion cultures to predict upfront critical DP characteristics such as gene transfer into LT-HSCs.

Iterative optimization of mPB CD34 cell expansion for gene-therapy applications

To further optimize the EX-U protocol for gene-therapy applications, we took an iterative approach. First, we asked whether IL-6 could be substituted with IL-3 based on receptor expression in cultured HSCs (see Figure 2G). Culture in the presence of IL-3 increased expansion by >3-fold (n = 3 volunteer mPB donors) while reducing the proportion of CD34$^+$ cells, leading, nevertheless, to a net gain in the absolute number of CD34$^+$ cells (Figures 3A and 3B). On the other hand, IL-6 tended to increase the absolute number of more primitive CD34$^+$CD90$^+$ cells (Figure 3C), and this effect was maintained when combined with intermediate doses of IL-3 (condition G, SFT63 combo). Linear mixed-effects models showed significant main effects of IL-3 and IL-6 on all three readouts ($p < 0.05$) and a significant IL-3 $\times$ IL-6 interaction for fold expansion (p = 0.005) reflecting a dominant IL-3 proliferative effect and a stronger IL-6-driven proliferative effect in the absence of IL-3 (Table S8). Xenotransplantation confirmed a trend for improved engraftment upon EX-U with SFT63, suggesting that intermediate doses of both IL-6 and IL-3 are beneficial (Figure 3D).

Second, EX-U was tested with SFT63 on mPB CD34$^+$ cells from n = 4 volunteer donors in the context of different commercial media. While fold expansion was similar, medium A (SCGM, CellGenix) best maintained cells with a primitive immunophenotype in culture, which translated into significantly higher engraftment as compared to medium B (Figures 3E and 3F). We then benchmarked our medium A/SFT63 condition against a chemically defined (ChemD) culture medium, which has recently been advocated for the expansion of CB HSCs.[23] Importantly, ChemD was unable to expand mPB HSPCs from n = 2 adult donors (Figure 3G). Expansion of CD34$^+$ cells was partially rescued when IL-3 was added to ChemD, confirming the importance of this cytokine for mPB cultures.

Third, we tested the addition of other small-molecule compounds, which have been shown to promote HSC expansion, to the EX-U condition. CD34$^+$ cells from mPB were transduced with the LVBC and expanded in medium A with SFT63 and UM171 (EX-U) or UM171 plus StemRegenin1 (SR1), an aryl hydrocarbon receptor antagonist (EX-US) (Figure 3H). Xenotransplantation showed no statistically significant differences in human CD45$^+$ cell engraftment of the EX-US condition compared to EX-U in the blood (Figure 3I) and the BM (Figure 3J), and neither in terms of clonal graft diversity

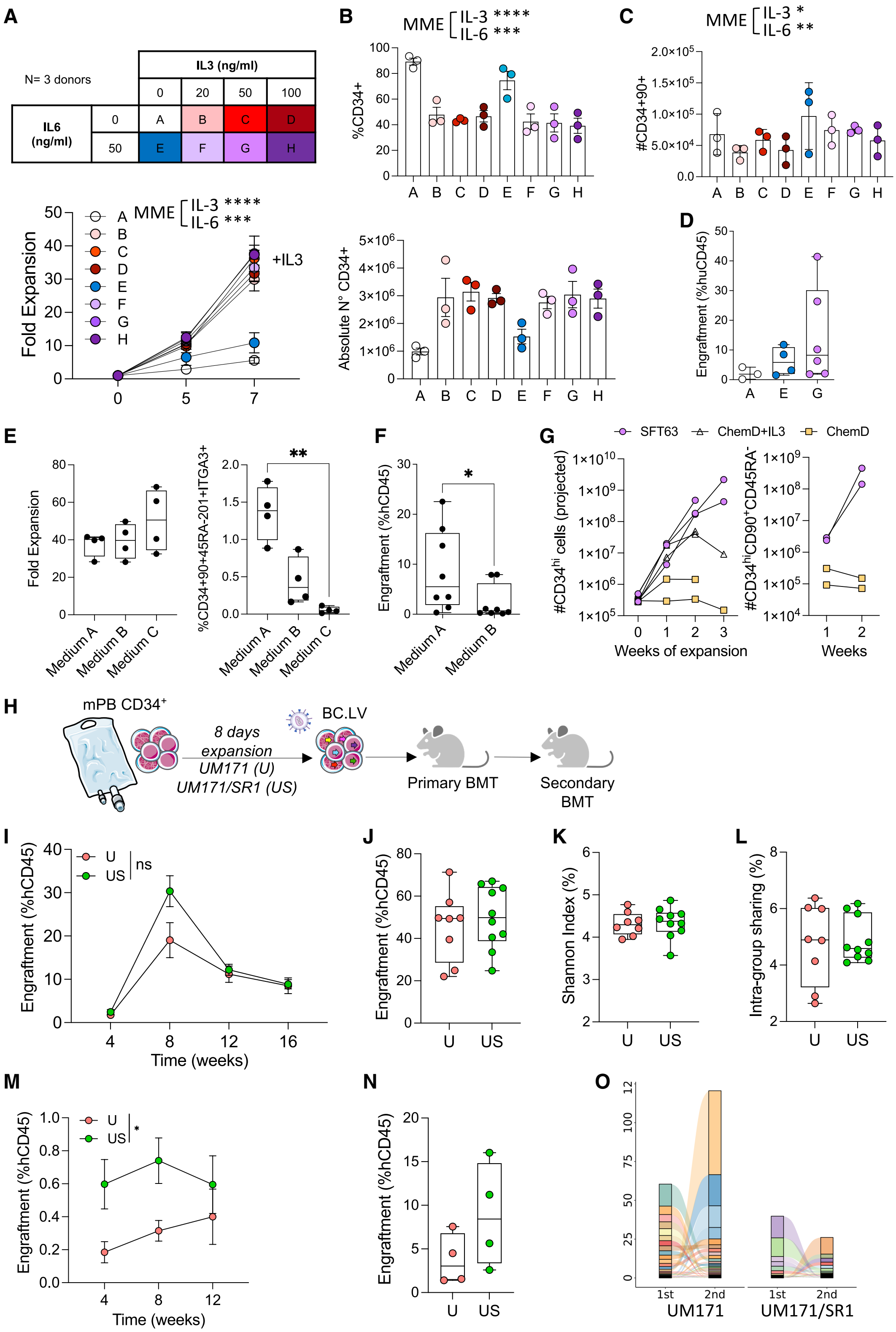

(legend on next page)

(Figure 3K; Table S4) or barcode sharing between mice (Figure 3L; Table S4), suggesting that EX-U and EX-US were similarly permissive to symmetric self-renewal divisions in culture. However, secondary transplantation showed significantly higher engraftment in the blood (Figure 3M) and a trend for higher BM engraftment (Figure 3N) in the EX-US group. To better understand the dynamics of secondary repopulation, we analyzed the clonal abundance of shared clones between the primary and secondary grafts (Figure 3O; Table S5). In both EX-U and EX-US conditions, the major clones contributing to hematopoiesis in secondary mice were initially minor clones in the primary grafts, and vice versa, confirming that these assays are reading out different waves of hematopoiesis driven by LT-HSC with different degrees of latency. Thus, SR1, though not critical when using optimized culture conditions, may provide additional benefits in maintaining LT-HSC with latency characteristics in culture.

Fourth, we investigated the use of the DEGS1 inhibitor, N-(4-hydroxyphenyl)retinamid (4HPR), in *ex vivo* expansion protocols. In line with previous reports,[21] we confirmed that 4HPR increased the *in vitro* clonogenic output of CB HSC ($CD34^{+}CD38^{-}CD90^{+}CD45RA^{-}CD49f^{+}$) but not of progenitors ($CD34^{+}$ $49f^{-}$) (Figure 4A). Enhanced CFU-forming potential was found also for sorted HSCs from mPB when supplemented with 4HPR irrespective of UM171 addition (Figure 4B), also when 4HPR was added after LV transduction, during a 7-day *ex vivo* expansion period prior to CFU seeding (Figure 4C). Encouraged by these data suggesting a specific impact of 4HPR on mPB HSCs, we expanded another DP from an MPS patient (Pt5) with the triple combination of UM171, SR1, and 4HPR (EX-US4) and compared engraftment to EX-US, EX-U, or the DP itself (Figure 4D). The EX-US4 condition showed significantly reduced BM engraftment as compared to EX-U at 12 weeks (Figure 4E) but no evident difference from the other conditions in secondary repopulation potential (Figure 4F). Clonal analysis by LVIS confirmed reduced number of unique integration sites in the 4HPR supplemented culture (Figure 4G) with no major changes in clonal size distribution of primary grafts (Figure 4H). We also confirmed no benefit from the addition of 4HPR to UM171 alone in the absence of SR1 (EX-U vs. EX-U4) for Pt1 and Pt2 xenografts (Figures S3C and S3D).

To understand whether LV transduction had a role in this unexpected outcome, we performed limiting dilution xenotransplantation experiments on mPB $CD34^{+}CD38^{-}$ cells from day-8 expansion cultures with the EX-US or EX-US4 condition, following mock- or LV transduction (Figures 4I and 4J). HSC estimates were 1/8,000 for EX-US and 1/4,200 for EX-US4 (p = 0.3, chi-squared test) in the mock groups when calculated on the day-0-equivalent dose.

Figure 3. Expansion protocol optimization for mPB HSPC gene-therapy applications

(A) (Top) The matrix outlines the IL-6 and IL-3 doses (ng/mL) used in the different culture combinations (identified by the letters A–H), tested on three healthy donors. All conditions contained SCF 100 ng/mL; FLT3L 100 ng/mL; TPO 50 ng/mL. (Bottom) Fold expansion over time (mean ± SEM) from three donors cultured with the different IL-3 and IL-6 concentrations. (B) The percentage (top) and absolute number (bottom) of $CD34^{+}$ cells after 7 days of expansion. Bars represent the mean ± SEM, individual donor values are shown as dots. (C) Absolute number (#) of $CD34^{+}CD90^{+}$ cells after 7 days of expansion . Bars represent the mean ± SEM, individual donor values are shown as dots. (A–C) Linear mixed-effects models with fixed effects: IL-3, IL-6, IL-3 × IL-6 and random effect for donor; log2-scale for fold expansion and absolute output, raw scale for %$CD34^{+}$. Type III ANOVA (Satterthwaite); *post hoc* Holm-adjusted contrasts. See materials and methods. (D) Percentage of human $CD45^{+}$ cells present in the BM of mice transplanted (n = 3–6) with cells derived from the indicated culture conditions 17 weeks post transplant. The mice were transplanted using a pooled sample from the three donors for each condition, each mouse receiving a limiting dose of 35,000–39,000 day-0-equivalent cells (Kruskal-Wallis test). (E) Different commercial media were compared (A, SCGM Cell Genix; B, StemSpan StemCell Technologies; C, StemPro GIBCO). Boxplot (left) showing the *in vitro* fold expansion measured after 7 days of culture of mPB $CD34^{+}$ cells from four different healthy donors. In all tested conditions, the culture media were supplemented with the same cytokine cocktail (100-ng/mL SCF, 100-ng/mL FLT3L, 50-ng/mL TPO, 50-ng/mL IL-6, 50-ng/mL IL-3). The percentage of primitive $CD34^{+}CD90^{+}CD45RA^{-}CD201^{+}ITGA3^{+}$ cells (right) was measured by flow cytometry after 7 days of expansion in different media. Each dot represents an individual donor (Kruskal-Wallis test with Dunn's multiple comparison, $^{*}p < 0.05$, $^{**}p < 0.01$). (F) Boxplot showing the percentage of human $CD45^{+}$ cells engraftment in the BM of mice transplanted (n = 8) with cells cultured in medium A or medium B at 16 weeks post transplant. Each dot represents a single mouse; mice were transplanted using a pool from different donors for each condition, with 48,000–57,000 day-0-equivalent cells injected per mouse (Mann-Whitney test, $^{*}p < 0.05$). (G) $CD34^{+}$ cells from n = 3 adult mPB donors were expanded over 3 weeks in medium A supplemented with SFT63 cytokines, UM729 and SR1 (SFT63), or a chemically defined medium (ChemD; see materials and methods) with or without IL-3. The projected absolute number (#) of $CD34^{high}$ cells (left) and more primitive $CD34^{hi}CD90^{+}CD45RA^{-}$ cells (right) over a 3-week culture period is shown, taking into consideration the proportion of cells that has been removed during consecutive passages. Connected dots refer to longitudinal data from an individual donor and the indicated condition. (H) Schematic representation of the experimental design. Different compound combinations were tested: UM171 (35 nM) alone (U) or in combination with SR1 (US) (750 nM). In both conditions, cells were cultured in SCGM with the optimized cytokine cocktail described above. (I) Percentage of human $CD45^{+}$ cells (mean ± SEM) present in the peripheral blood (PB) of U (n = 8) and US (n = 10) conditions at the indicated number of weeks post transplant. ns, not significant (U vs. US, mixed-effects analysis). (J) Percentage of human $CD45^{+}$ cells present in the BM at 17 weeks post transplant. (K) Box plots indicating the Shannon diversity index as measure of clonal population diversity. Each dot indicates an individual mouse of the U and US primary recipient groups. (L) Sharing of barcodes within the same treatment group, measured as the percentage of shared barcodes from individual mice (dots) of the same treatment group. Each dot represents the percentage of LVBCs shared with at least one other mouse in the same experimental group. (M) Percentage of human $CD45^{+}$ cells (mean ± SEM) present in the peripheral blood (PB) of U and US (n = 4) conditions at the indicated number of weeks post-secondary transplant. The mice were transplanted using a pooled sample from BM of primary recipients depleted of murine cells. The amount of $CD34^{+}$ cells transplanted into secondary recipients ranged between 1–1.3 × 10^{6} $CD34^{+}$ cells. $^{*}p < 0.05$ (U vs. US, two-way ANOVA). (N) Boxplot with percentage of human $CD45^{+}$ cells present in the BM of mice transplanted (n = 4) with cells derived from primary recipients, 17 weeks post secondary transplant. (O) Alluvial plots showing the frequency of shared barcodes between primary and secondary transplants. Barcode frequencies are arranged from most abundant to least abundant within the primary transplant recipients' BM, only shared barcodes across primary and secondary transplant recipients are plotted. Barcode frequency is calculated as the mean abundance of the barcode across mice from the same experimental condition in which the barcode could be detected. Left and right alluvial plots show the sharing in the UM171 (U) and UM171/SR1 (US) conditions, respectively.

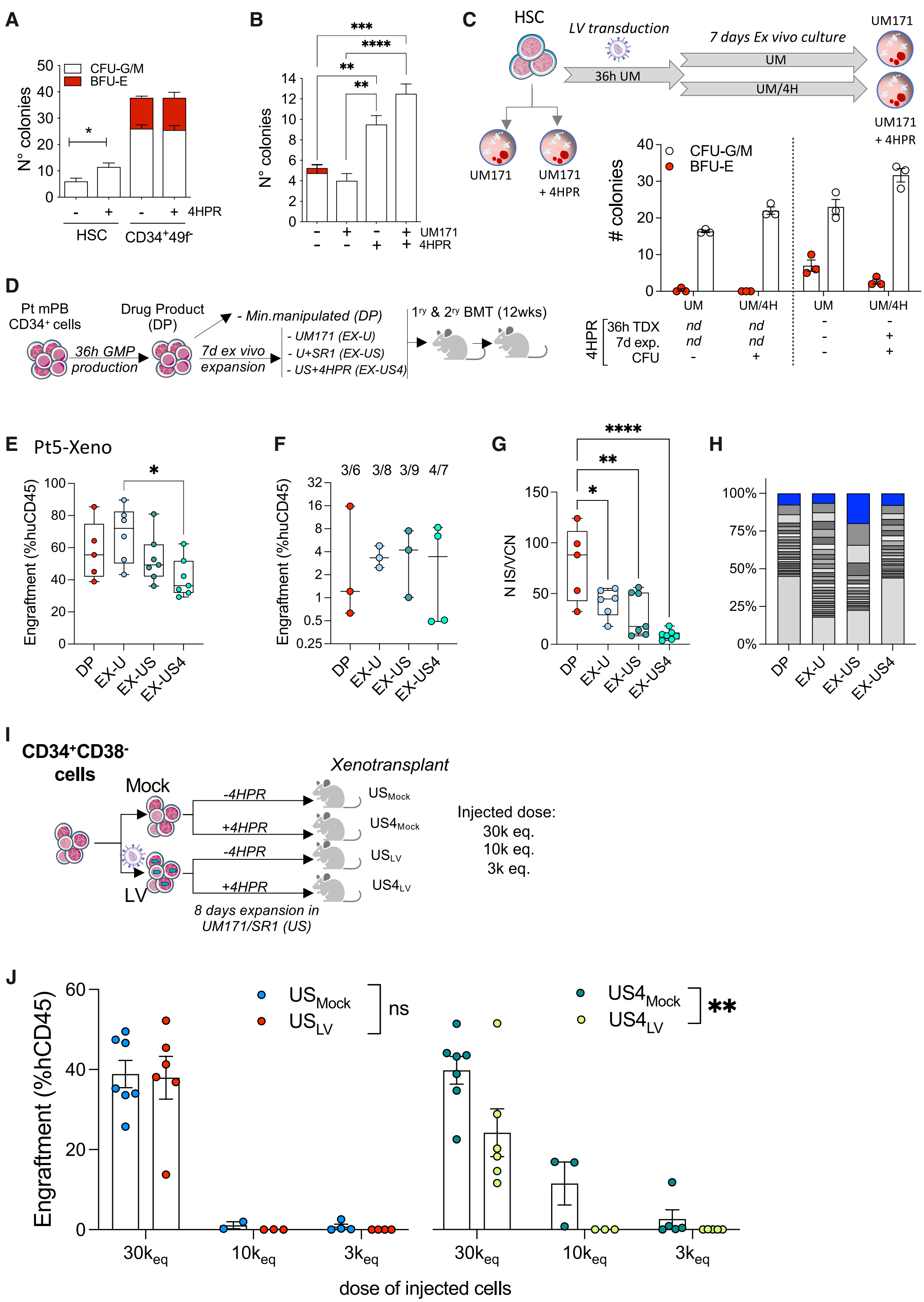

(legend on next page)

However, LV transduction significantly decreased HSC day-0-equivalent frequency to approximately 1/18,000 cells in both the EX-US and EX-US4 conditions ($p < 0.03$, chi-squared test) (Figure S3E).

Taken together, these data suggest that LV transduction cumulatively adds to a stress response in the *ex vivo* expansion setting, which may render HSC functionally deficient in xenotransplantation assays. Combination of proteostasis inhibition with LV transduction is thus not considered beneficial for mPB-HSC gene-therapy protocols.

Controlling dose and transduction time window alleviate LV toxicity in the *ex vivo* expansion setting

Considering the unexpected sensitivity of *ex vivo* expansion cultures to LV transduction, we further investigated this critical genetic-engineering step to evaluate conditions that could mitigate LV-induced toxicity and better preserve engraftment potential by shifting the LV exposure time window from the canonical time window of 24–48 h after the start of *ex vivo* culture by 24-h increments (Figure 5A). *In vitro* maintenance of CD34 expression was consistent across all conditions (Figure 5B), while longer pre-stimulation increased overall transduction efficiency (Figure 5C). However, engraftment potential of EX-U cells dropped sharply when transduction was carried out in the 72- to 96-h window (late transduction group, EX-U3), while it was equal or slightly superior to DP_{like} cells if transduction was conducted before (Figure 5D). Analysis of intragroup barcode sharing confirmed symmetric *ex vivo* HSC division, with a trend for fewer shared barcodes in the late-transduction group compared to EX-U2 (Figures 5E; Table S6). To gain further insight into the mechanism behind LV-induced loss of HSC potency, we performed bulk RNA sequencing on *ex vivo*-expanded HSPCs transduced at early (24-h) or late (72-h) time points. $CD34^+$ HSPCs exposed to the LV during the 72- to 96-h window of *ex vivo* expansion showed strong enrichment of senescence- and aging-associated pathways, adding to the evidence that LV transduction may be detrimental in the context of *ex vivo* expansion in a dose- and time-dependent manner (Table S7; Figure 5F). Based on these findings, we selected an intermediate transduction time window from 36 to 48 h (EX-U2) after starting pre-stimulation for further testing.

We benchmarked this optimized transduction/expansion protocol (EX) against a clinically validated short manufacturing protocol (DP_{like}) for gene therapy (Figures 5G and 5H).[30,34] The comparison was conducted using a good manufacturing practice-compliant, large-scale process run in gas-permeable bags and a therapeutically relevant, purified LV vector, developed for the treatment of autosomal recessive osteopetrosis, a setup suitable for clinical translation.[34] Engraftment of DP_{like} (2×10^5 cells/mouse) was compared to a low or high dose of EX, chosen to mimic clinically validated human transplant doses (EX_{low}, 4×10^6 and EX_{high}, 28×10^6 cells/mouse, corresponding to a day-0 equivalent of 1×10^5 and 7×10^5 cells/mouse or 5×10^6 to 35×10^6 cells/kg, respectively; $n = 14$ per cohort). Multilineage human cell engraftment in the blood at 8 weeks post transplantation and in the BM at 19 weeks (Figure 5I) was highest for the EX_{high} group, while the DP_{like} group positioned in between EX_{high} and EX_{low}. These data align with a net HSC maintenance during *ex vivo* culture when considering the overall graft. However, engraftment of genetically engineered cells, which are the therapeutically relevant part of the graft, was higher in EX as compared to DP_{like}, even in the EX_{low} group, as shown by an increased VCN per human genome (Figure 5J), an increased absolute number of unique integration sites normalized to VCN (Figure 5K), and an increased Shannon diversity index (Figure 5L). These results highlight an advantage of the optimized expansion protocol over the current standard, even at lower transplant cell doses.

DISCUSSION

Efforts to optimize *ex vivo* HSC manipulation protocols have mainly focused on minimizing culture time to preserve repopulating potential, reduce clonal loss, and mitigate senescence or genomic

Figure 4. Cumulative toxicity between proteostatic cellular stress and lentiviral transduction

(A) Colony count in CFU assays with sorted HSCs ($CD34^+CD38^-CD90^+CD45RA^-CD49f^+$) or $CD34^+$ ($CD34^+$ $49f^-$) cells (pool of $n = 4$ CB donors) in presence (+) or absence (−) of 4HPR (2 μM). The mean ± SEM of four replicates (600 cells/plate) is shown (unpaired *t* test). (B) Colony count in CFU assays with sorted HSCs ($n = 1$ mPB donor) in presence (+) or absence (−) of 4HPR (2 μM) and or UM171 (35nM). The mean ± SEM of four replicates (800 cells/plate) is shown (one-way ANOVA with Bonferroni correction). (C) (Top) Experimental design. Expansion culture and/or colony assays were conducted with UM171 (UM) in presence (+) or absence (−) of 4HPR (4H). (Bottom) Colony count in CFU assays with sorted HSC ($n = 1$ new mPB donor) performed either on freshly sorted cells (two groups on the left) or after LV transduction and *ex vivo* expansion (two groups on the right), in the presence (+) or absence (−) of 4HPR (2 μM), as shown at the bottom of the graph (nd, not done). The mean ± SEM of three replicates (1,000 cells/plate) is shown. (D) Schematic representation of xenotransplantation experiments performed on the DP from an MPS patient (Pt5). (E) Human cell engraftment in the BM of NSG mice at 12 weeks after transplantation of (1) 200,000 cells from the drug product (DP) ($n = 5$); (2) 5,400,000 *ex vivo*-expanded cells EX-U (equivalent to 200,000 $CD34^+$ cells at day 0, expanded for 7 days with UM171; $n = 6$); (3) 6,200,000 *ex vivo*-expanded cells EX-US (equivalent to 200,000 $CD34^+$ cells at day 0, expanded for 7 days with UM171 and SR1; $n = 7$); (4) 1,300,000 *ex vivo*-expanded cells EX-US4 (equivalent to 200,000 $CD34^+$ cells at day 0, expanded for 7 days with UM171, SR1, and 4HPR; $n = 7$); Kruskal-Wallis test with Dunn's multiple comparison, $^*p < 0.05$. (F) Human cell engraftment in the BM, 15 weeks after secondary transplantation of 1×10^6 $CD34^+$ cells per mouse, isolated from primary recipients described in (E). The number of mice with multilineage engraftment over the total number of transplanted mice is shown. (G) Box plots indicating the number of unique integration sites normalized on VCN retrieved from the BM of primary grafts described in (E). One-way ANOVA with Tukey's multiple comparison test. (H) Relative abundance of integration sites in the primary recipient mice. Bottom light-gray stacks indicate the sum of integrations with <1% relative abundance, middle stacks (shades of gray) indicate integrations >1% abundance, and the top abundant integrations highlighted in blue. (I) Schematic representation of xenograft experiments performed on the progeny of sorted $CD34^+CD38^-$ cells from adult mPB testing the following 2 variables: (1) transduction (LV) or not (mock) with a purified LV vector; (2) expansion culture with or without 4HPR. (J) Human cell engraftment in the BM was evaluated by analyzing the proportion of $CD45^+$ cells 16 weeks post injection. Shown is the mean ± SEM; each dot represents an individual mouse. A mouse was classified as engrafted if it contained >0.5% of $hCD45^+$ cells. ns, not significant; $^{**}p < 0.01$ (mock vs. LV, two-way ANOVA).

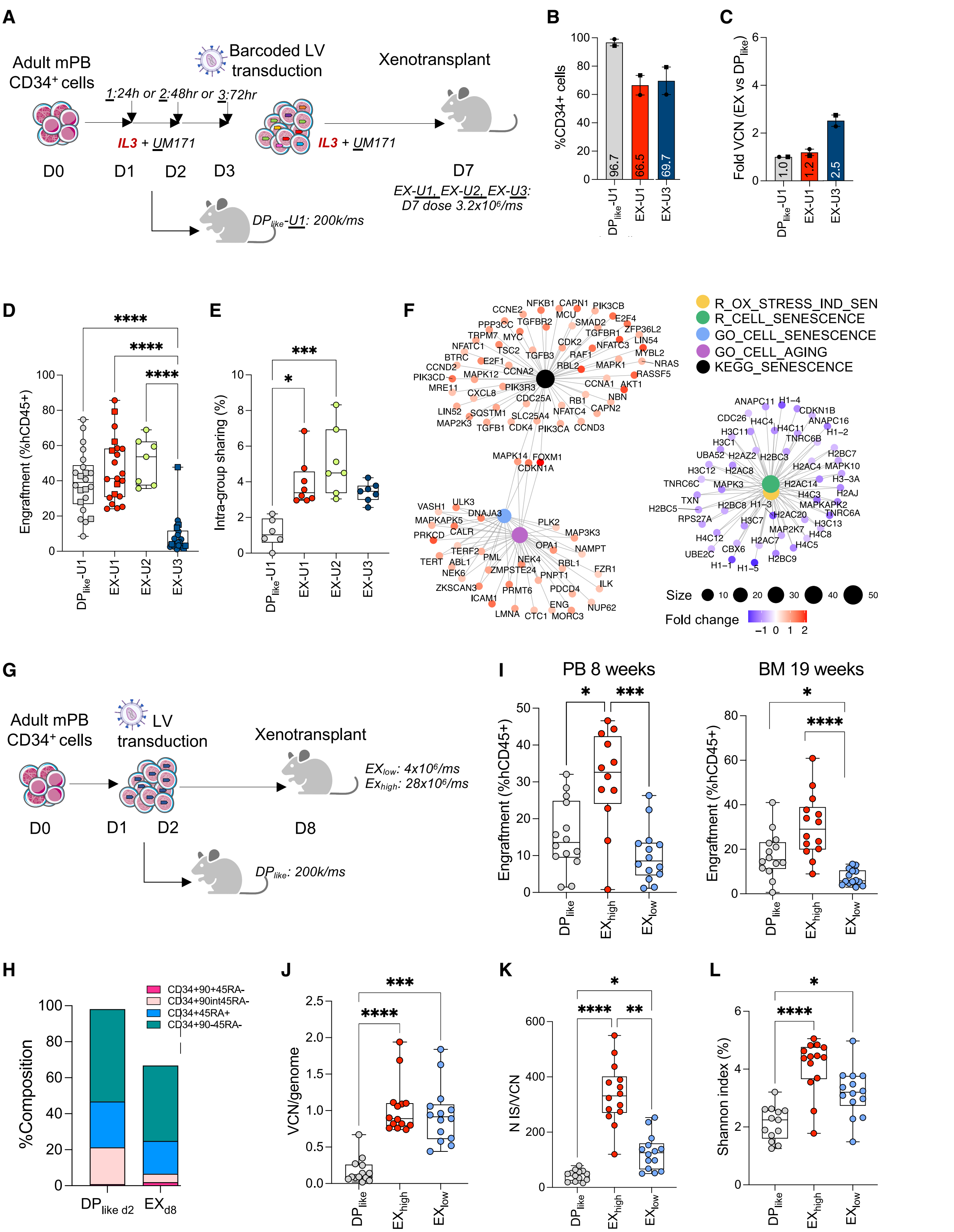

(legend on next page)

instability. The inclusion of molecules that antagonize undesired culture effects and the careful protocol optimization we have undertaken have made it possible to extend *ex vivo* culture time of mPB to at least a week without losing HSC function or clonality compared to state-of-the art short-culture protocols. Not surprisingly, the degree of net HSC expansion was lower in mPB as compared to published CB studies,[12,18] which may be explained by higher functional HSC content[35] and faster cell cycle kinetics[36] in the latter. Nevertheless, expanded mPB products exhibited potent multilineage engraftment potential, higher VCNs per genome, and preserved clonal diversity even at lower transplant doses compared to shortly cultured cells. Thus, *ex vivo* expansion of mPB HSCs represents a promising avenue for overcoming limitations in cell availability and enhancing therapeutic efficacy for gene-therapy applications.

We present the stepwise optimization of a clinically translatable, good manufacturing practice-compliant expansion protocol tailored for mPB-derived HSCs from both adult and pediatric donors. We optimized cytokine combinations by exploiting predicted receptor expression in scRNA-seq identifying a benefit in adding intermediate doses of IL-3 to promote the growth of progenitors while maintaining the absolute number of phenotypic HSCs, potentially by stimulating asymmetric HSC division. Even though the receptor for IL-6 does not appear to be highly expressed on HSCs, intermediate doses of IL-6 increased the number of phenotypic HSCs. This may potentially be due to non-cell autonomous effects, e.g., by stimulating crosstalk between monocyte precursors, on which the IL-6 receptor is upregulated, and HSCs, a phenomenon that has been described to enhance engraftment potential.[37] Often overlooked in protocol design, medium selection significantly influenced the yield of functional HSCs as defined by xenograft assays, a cumbersome readout generally not performed by developers of commercial cell-culture media. Lack of transparency on formulations and single ingredients preclude identification of the beneficial and harmful components contained in the different commercial products. Lastly, we explored combinations of HSCs maintaining molecules on top of UM171, which, in our hands[31] and in the hands of others,[12,23] has been the most potent compound. Our data now show an added benefit of blocking the aryl hydrocarbon receptor pathway by SR1, most notably on LT-HSCs when challenged in secondary repopulation assays or under non-optimal *ex vivo* expansion conditions (data not shown). We observed toxicity from the addition of 4HPR to mPB HSCs in our protocol, aggravated by LV transduction. While the exact mechanism of this toxicity remains to be investigated, we may speculate that mPB HSCs, by themselves poised to an increased basal pro-inflammatory state,[38,39] which is further enhanced by cytokine pre-stimulation and LV exposure, may exhaust compensatory mechanisms when challenged with 4HPR-induced oxidative/ER stress.[21]

An unexpected finding was also the influence of the timing of LV transduction on HSC functionality. The response of human HSPCs to exposure of purified LVs has been described as rather limited, with some degree of p53 activation through nuclear sensing of vector DNA but little evidence for innate immune activation.[33] This is in line with clinical data whereby even highly transduced DPs engrafted promptly and did not cause measurable side effects in the context of a short *ex vivo* culture protocol.[30] Interestingly, the sensitive time window of LV transduction coincides with the first cell division of purified mPB LT-HSCs, estimated to occur around 62.0 ± 6.5 h,[11] which may increase the susceptibility of HSCs to undergo senescence upon p53 activation. Alternatively, the higher VCN obtained from longer pre-stimulation times may also contribute to the observed reduction in engraftment potential. These findings require follow up studies but place special attention to HSC manipulations when performing prolonged *ex vivo* HSC cultures, where cells are exposed to metabolic activation and proliferative stress. Notably, a recent study has found a very similar vulnerability time

Figure 5. Controlling dose and transduction time window alleviates LV toxicity in the *ex vivo* expansion setting

(A) Schematic representation of the experimental design for data shown in (B)–(F). Two separate transplant sessions were performed using $CD34^+$ cells from the same adult mPB donor under the same protocol; each session transplanted cell products from all groups, except the EX-U2 condition, which was added during the second transplant session. Pooled results from the two sessions are shown. (B) The percentage of $CD34^+$ cells measured by flow cytometry after 7 days of expansion under different conditions. Bars represent the average from the two experimental sessions. Data not shown for EX-U2 (76%) as only a single replicate was performed. (C) Relative VCN change in the EX-U condition with respect to DP_{like}, as determined by ddPCR on liquid culture ($n = 2$ experimental sessions; $n = 1$ for EX-U2 [4.8], data not shown). (D) Engraftment potential of mPB $CD34^+$ cells in NSG mice transplanted with either (1) 200,000 cells from the DP_{like}-U1 condition ($n = 22$) or (2) 3,200,000 *ex vivo*-expanded cells (equivalent to 200,000 $CD34^+$ cells at day 0) expanded for 7 days and transduced at the following time points: EX-U1, 24 h, $n = 21$; EX-U2, 48 h, $n = 7$; EX-U3, 72 h, $n = 22$. Human cell engraftment in the BM was evaluated by analyzing the proportion of $CD45^+$ cells 17 weeks post injection (Kruskal-Wallis test with Dunn's multiple comparison). (E) Intragroup LVBC sharing percentages. Each dot represents the percentage of sharing between an individual mouse vs. all other mice receiving the same HSPC product (Kruskal-Wallis test with Dunn's multiple comparison, $^*p < 0.05$, $^{***}p < 0.001$, $^{****}p < 0.0001$). (F) Bulk RNA sequencing was performed to identify DEGs between the EX-U3 and EX-U1 groups. The cnet plot shows enriched senescence terms obtained from GSEA; blue and red dot colors represent down-/upregulated genes, respectively. The dot sizes represent the number of genes that belong to the term. (G) Experimental scheme. Adult mPB $CD34^+$ cells were transduced with a clinical-grade LV and either expanded (EX) or not (DP_{like}) using a good manufacturing practice-compliant process. The following groups were injected into NSG mice: (1) 200,000 DP_{like} cells ($n = 14$), (2) 20,000,000 *ex vivo*-expanded cells at high-dose EX_{high} (equivalent to 530,000 $CD34^+$ cells at day 0; $n = 12$), or (3) 4,000,000 *ex vivo*-expanded cells low-dose EX_{low} (equivalent to 100,000 $CD34^+$ cells at day 0; $n = 14$). (H) Percentage of $CD34^+$ cells and subset composition measured by flow cytometry in the respective products (day 2, DP_{like}; day 8, EX) before xenotransplantation. (I) Human $CD45^+$ cell engraftment measured in the peripheral blood (left) and BM (right) at the indicated time points (Kruskal-Wallis test with Dunn's multiple comparison). (J) VCN determined by ddPCR on BM cells from mice of different groups (Kruskal-Wallis test with Dunn's multiple comparison). (K) Box plots indicating number of unique integration sites normalized on VCN (Kruskal-Wallis test with Dunn's multiple comparison). Each dot represents an individual mouse (Kruskal-Wallis test with Dunn's multiple comparison). (L) Box plots indicating the Shannon diversity index as a measure of clonal population diversity, (Kruskal-Wallis test with Dunn's multiple comparison). Each dot indicates an individual mouse.

window of HSCs to gene editing triggering a DNA damage response, which could be mitigated by slowing cell-cycle progression through p38 mitogen-activated protein kinase (MAPK) inhibition.[40]

A major challenge in successful development of *ex vivo* expansion protocols is accurately predicting *in vivo* repopulating capacity based on *in vitro* product characteristics. Many validated surface HSC marker profiles, such as CD38, lose their predictive value once cells are manipulated *ex vivo*, or they may not be consistently expressed across cell sources.[31,41,42] We thus undertook an unbiased characterization of *ex vivo*-cultured HSPCs by scRNA-seq, which provided high-resolution insights into cellular heterogeneity and lineage fidelity of committed precursors during culture. Additionally, scRNA-seq enabled predictive analyses confined to functionally relevant, select cell populations, namely molecularly defined HSCs, in which *in vitro* VCN prediction correlated with the VCN measured in xenografts and, importantly, with the VCN reported in patients following LT engraftment.[30] Expansion culture was required to accurately assess transduction efficiency in HSCs, since direct analysis of the DP may yield false-positive results, presumably due to the presence of transcripts from non-integrated vector forms. Hence, this provides a proof-of-concept that *ex vivo* expansion cultures may serve as a cost-effective surrogate for *in vivo* cell product dynamics assessment, which may eventually replace xenograft experiments, even though additional validation on a higher number of samples is needed.

Clonal tracking analyses stringently validated our *ex vivo* culture by assessing for the presence of symmetric self-renewal divisions *ex vivo* with increased sharing of genetic marks across mice transplanted from expansion culture as well as ruling out clonal dominance or oligo-clonal selection—a critical safety consideration in gene-therapy applications. A recent study has used barcoding to show self-renewal of molecularly defined HSCs (by scRNA-seq) during *in vitro* culture of CB in the presence of UM171.[43] We add to this conclusion by demonstrating self-renewal of functionally defined HSCs derived from mPB. Integrating scRNA-seq-based identification of HSC transcriptional states with *in vivo* clonal tracking by scRNA-seq-compatible barcoded LV libraries could yield even deeper insights into cellular states and dynamics during *ex vivo* expansion and related *in vivo* implications. Our clonal bulk analysis *in vivo* revealed distinct waves of hematopoietic repopulation between primary and secondary xenografts, mirroring what has been previously described as the output of latent LT-HSC subsets resistant to minor stress-induced activation.[44] These latent $CD112^{lo}$ LT-HSCs are supposed to preserve self-renewal capacity by remaining quiescent under stress conditions but are recruited during major regenerative demands such as those imposed by a secondary transplantation. Nevertheless, our data suggest that these latent HSCs have a memory of the *ex vivo* culture, as suggested by improved secondary reconstitution of HSCs exposed to the combination of SR1 and UM171. The ability to show polyclonal engraftment in secondary transplants with our protocol is again a strong indicator of preservation of *bona fide* HSC functionality with our optimized expansion protocol.

Despite our in-depth functional and molecular characterization of *ex vivo* mPB expansion cultures down to single-cell/single clone resolution, and systematic process optimization, we could not obtain a consistently high expansion of mPB LT-HSC numbers, which may in part be explained by distinct biological properties with respect to CB. Two approaches can be envisioned to further increase net HSC expansion: (1) stimulate HSC self-renewal divisions, by targeted molecular interventions in the self-renewal program[45] or, most simply, by increasing the length of culture considering that slow cell-cycle entry and progression is a core property of LT-HSCs.[46] (2) Mitigate the loss of HSCs through irreversible differentiation, e.g., by reducing culture stress through inhibition of pathways such as JAK,[11] MAPK,[40] or mTORC[10]; titration of oxygen levels[47]; and mechanoregulation.[48] It should be highlighted that our functional readouts concentrate on HSCs and do not assess early hematopoietic reconstitution driven by progenitors, which cannot be read out in the NSG model and has not been further evaluated in this work. A clinical implication of limited net LT-HSC expansion from mPB is that, unlike CB-based protocols, the starting cell requirement may not be dramatically reduced, and adequate mobilization remains essential. Nevertheless, the substantial amplification of committed progenitors during expansion is expected to shorten the duration of post-transplant cytopenia. Hence, *ex vivo* mPB expansion with our optimized protocol is expected to offer clinical benefit in terms of myeloid and platelet reconstitution over NC or minimally cultured cells, pending confirmation in future clinical trials. Notably, we have submitted a clinical trial application for a phase I/IIa gene-therapy study (EU CT 2024-518972-30) in children affected by autosomal recessive osteopetrosis (ARO), a rare and severe genetic bone disorder characterized by defective osteoclast function and the lack of a BM HSC niche, where rapid reconstitution of resorption-competent osteoclasts may allow proficient engraftment of corrected LT-HSCs.[49]

Importantly, our optimized expansion protocol is compatible with existing good manufacturing practice-compliant workflows and standard release testing, including viability, immunophenotype, sterility, and VCN assessment. However, extended culture and additional reagents may increase operational complexity and costs. Scalable implementation using automated or modular expansion systems is feasible, and the use of clinically validated materials ensures alignment with current regulatory frameworks. Overall, these features support both the practical integration of the protocol into manufacturing pipelines and its potential broader clinical application where further disease-specific optimizations may be implemented. In sum, we have developed an optimized *ex vivo* mPB expansion protocol to aid in genetic correction of *ex vivo*-expanded autologous circulating HSCs to address unmet medical needs.[34]

MATERIALS AND METHODS

Human HSPCs

Human CB and mPB $CD34^+$ HSCs were purchased from commercial sources (Lonza, HemaCare, All Cells, and Charles River). Aliquots of DPs from patients enrolled in the TigetT10_MPSIH trial

Table 1. List of anti-human antibodies used for flow cytometry

Antibody	Fluorochrome	Clone	Company	Code
FcR blocking reagent	–	–	Miltenyi Biotec	130-059-901
CD45	APC-eFluor780	HI30	eBioscience	47-0459-42
CD45	VioBlue	REA747	Miltenyi	170-081-077
CD19	PE-Cy7	HIB19	BioLegend	302216
CD19	PE	SJ25C1	BD Biosciences	345789
CD3	PE	SK7	BD Biosciences	345765
CD3	PB	UCHT1	BioLegend	300431
CD13	APC	WM15	BD Biosciences	557454
CD33	APC	AC104.3E3	Miltenyi Biotec	130-091-731
CD33	PE-Cy7	P67.6	BD Biosciences	333952
CD33	APC-Vio770	REA775	Miltenyi Biotec	130-111-022
CD34	VioBlue	AC136	Miltenyi Biotec	130-095-393
CD34	PE-Cy7	8G12	BD Biosciences	348811
CD34	APC	AC136	Miltenyi Biotec	130-120-519
CD34	BV421	561	BioLegend	343610
CD34	PE	AC136	Miltenyi Biotec	130-113-179
CD38	PE-Vio770	IB6	Miltenyi Biotec	130-099-151
CD90	APC	5E10	BD Biosciences	559869
CD90	BV421	5E10	BD Biosciences	562556
CD45RA	PE	T6D11	Miltenyi Biotec	130-092-248
CD45RA	FITC	HI100	BioLegend	304106
CD56	PE	NCAM 16.2	BD Biosciences	345812
BAH1	PE	BAH-1	BD Biosciences	565746
CD71	BV711	M-A712	BD Biosciences	563767
CD49f	PC5	GoH3	BD Biosciences	562495
7AAD	–	–	Miltenyi Biotec	130-111-568
Zombie Aqua™	–	–	BioLegend	423101
P16 INK4A	–	D7C1M rabbit mAb	Cell Signaling Technology	80772S
Alexa Fluor 488- labeled secondary antibodies	–	–	Thermo Fisher Scientific	A-21206
MitoTracker™ Green FM Dye	–	–	Thermo Fisher Scientific	M46750

mAb, monoclonal antibody.

(NCT03488394, see Gentner et al.[30] for relevant design details) and specifically allocated for research purposes were available in accordance with the clinical trial protocol and the associated research protocols (TIGET09), which have been approved by the responsible ethical committees. Informed consent for the use of these materials in research was obtained from all patients' legal guardians.

Sample size and replicates

Sample size pre-determination was used for the experiment validating the optimized expansion culture setup (Figures 5G–5L). For the experiments with patient cells, exploratory experiments and iterative process optimization steps, sample size for each experiment was dependent on the total number of available HSPCs, which is constrained by the human source of the material and limited by transduction efficiency, scale, and recovery from cell expansion culture or primary recipient xenografts. Whenever possible, we aimed to reach $n \geq 5$ per group, which is considered adequate for carrying out nonparametric statistical comparisons.

Data were collected at prespecified endpoints for transplantation experiments, with primary recipients euthanized at 12 weeks post engraftment if secondary transplants were to be carried out or at longer time points (≥16 weeks). All samples passing prespecified quality-control metrics were included in the presented analyses.

Due to the limitations on animal experimentation, some exploratory experiments were performed once. If replicate experiments were conducted, they are specified within the specific figure legend.

Study design and research objective

We here show data from controlled laboratory experiments. Each experimental design is appropriately outlined in the text or figure and addresses a specific hypothesis for the improvement of *ex vivo* culture protocols for mPB HSPC expansion. When performing mouse experiments, mice were randomly allocated to the control or experimental arm. Experiments were not conducted in a blinded fashion, as blinding was not relevant for objective outcome measures.

HSPC culture, transduction, and expansion protocols

HSPC pre-stimulation and transduction was performed in serum-free medium at a cell density of 1×10^6 cells/mL. For *ex vivo* expansion culture, cell density was reduced to 2×10^5 cells/mL and maintained at $<1 \times 10^6$ cells/mL. Specific culture media and cytokine addition varied across experimental plan, as detailed in the results section and figure legends.

mPB HSPC DPs were cultured in SCGM medium (Cell Genix) supplemented with the following cytokines: 60-ng/mL IL-3, 100-ng/mL TPO, 300-ng/mL SCF, and 300-ng/mL FLT3L (all from CellGenix).[50,51]

Unless otherwise indicated, expansion cultures were established in serum-free SCGM CellGenix medium supplemented with 100 ng/mL SCF, 100 ng/mL FLT3L, 50 ng/mL TPO, 50 ng/mL IL-6, and 35 nM UM171 (Stem Cell Technologies or ExCellThera). In selected conditions, 750 nM SR1 (StemRegenin1; Stem Cell Technologies) and/or 2 μM 4HPR (a kind gift from the J.E. Dick laboratory) were also included. Cultures were maintained at 37°C in 5% CO2 under normoxic conditions.

A ChemD expansion medium was adapted from the literature[23,47] as follows: Iscove's modified Dulbecco's medium (IMDM) supplemented with Soluplus (BASF) 1 mg/mL, 740 Y-P (Cayman Chemical) 1 μM, Butyzamide (TargetMol) 0.1 μM, monothioglycerol (Sigma) 100μM, UM729 (Stem Cell Technologies) 1 μM, ITSX, Glutamax, and low-dose SCF and FLT3L (CellGenix), both 25 ng/mL. LV transduction was performed by addition of 10^8 transducing units/mL (multiplicity of infection 100, unless otherwise indicated) to the cell cultures for 16–24 h.

LV production, titration, and molecular analysis of gene-transfer efficiency

Purified vectors PGK.IDUA or TCIRG1 (Phosphoglycerate kinase promoter driving the alpha-L-iduronidase or T cell immune regulator 1 gene) LVs were produced and titered by MolMed (now AGC Biologics) according to the good manufacturing practice process employed for gene-therapy clinical trials.[50,51] Lab-grade vectors, third-generation self-inactivating (SIN) LV (PGK.GFP, PGK.BFP, PGK.mCherry) expressing different fluorescent proteins under the control of a PGK promoter stocks were produced and titered on 293T cells according to standard lab protocols as previously described.[52] Large-scale LV barcode library was produced and purified by a specialized institutional process development lab as described in Soldi et al.[53] Molecular quantification of gene-transfer efficiency was performed using specific digital droplet PCR (ddPCR) assays.

Clonogenic assay on Methocult

Clonogenic assays were plated at the time point described for each single experiment. Cells were washed, counted, and resuspended in complete human Methocult medium (Stem Cell Technologies) at a concentration of 800–2,000 cells/mL. Fourteen days later, colonies were scored by light microscopy for number and morphology. CFU-E and BFU-E were scored as erythroid colonies; CFU-G, CFU-M, and CFU-GM as myeloid; and CFU-GEMM as mixed colonies. If necessary, at day 14, colonies were single-picked and analyzed by ddPCR to evaluate transduction efficiency.

Determination of VCN by ddPCR

Genomic (g)DNA was extracted using QIAmp DNA micro kit (Qiagen) or QIAmp DNA mini kit according to the starting number of cells (as suggested by manufacturer). DNA was quantified and assessed for purity. Vector copies per diploid genome (VCN) of the integrated LV vectors were quantified by ddPCR on a QX200 Droplet Digital PCR System (Bio-Rad) starting from 10 to 100 ng of template genomic DNA (gDNA) using the following primers (HIV sense, 5′-TACTGACGCTCTCGCACC-3′; HIV antisense, 5′-TCTCGAC GCAGGACTCG-3′), and probe (FAM 5′-ATCTCTCTCCTTCTA GCCTC-3′) against the primer-binding-site region of LVs. Endogenous DNA amount was quantified by a primer/probe set against the human telomerase gene (Telo sense, 5′-GGCACACGTGGCTT TTCG-3′; Telo antisense, 5′-GGTGAACCTCGTAAGTTTATGC AA-3′; Telo probe, VIC 5′-TCAGGACGTCGAGTGGACACG GTG-3′ TAMRA). Alternatively, the region of the primate transcription initiation factor TFIID subunit 7 gene (TAF7), which is conserved among primates and humans, was used as an endogenous control assay (sequence not disclosed). The VCN was determined by calculating the ratio of the target-molecule concentration to the reference-molecule concentration times the number of copies of reference species in the genome. All the reactions were performed according to the manufacturer's instructions and analyzed with a QX200 Droplet Digital PCR System (software: QuantaSoft Version1.7.4.0917; Bio-Rad).

Cell sorting and flow cytometry

Sorting for CD34$^+$ cells (and CD38 cells, where indicated) was performed by magnetic beads (Miltenyi). FACS for HSPC subpopulations was performed on a MoFlo XDP sorter (Beckman Coulter) or BD FACSAria Fusion (BD). Analytical flow cytometry was performed on a FACSCanto II or LSR II Fortessa instrument (BD Bioscience).

Immunophenotypic analyses were performed by flow cytometry using Canto II (BD Pharmingen). From 5.0×10^5 to 2.0×10^6 cells either from culture or mouse-derived samples were analyzed. Cells were stained for 15 min at 4°C with antibodies listed in Table 1, in

a final volume of 100 μL, and were washed with Dulbecco's Phosphate-Buffered Saline (DPBS) + 2% heat-inactivated fetal bovine serum (FBS). Single-stained and fluorescence-minus-one-stained cells were used as controls.

Senescence-associated-β-galactosidase analysis

Senescence-associated-β-galactosidase analysis was performed with the fluorescent SPiDER-β-Gal system (Dojindo), as described.[54] Briefly, 1×10^5 cells were washed with 2% FBS in DPBS and incubated with 150 μM chloroquine (Sigma-Aldrich) for 1 h at 37°C. Then, SPiDER-β-Gal was added to the cell suspension at a final concentration of 0.57 ng/μL, together with anti-human CD34 BV421 (BioLegend, 1:100 dilution), anti-human CD90 APC (BD Biosciences, 1:100 dilution), and Zombie Aqua viability dye (BioLegend, 1:400 dilution) for 15 min at 37°C. Cells were washed with 2% FBS in DPBS, and samples were immediately acquired on a FACSCanto II (BD Biosciences). Data were analyzed using the FlowJo software.

p16 analysis

p16 analysis via flow cytometry was performed as recently described.[54] Briefly, 1×10^5 cells were washed with 2% FBS in DPBS and stained for 15 min at 4°C with anti-human CD34 BV421 (BioLegend, 1:100 dilution), anti-human CD90 APC (BD Biosciences, 1:100 dilution), and Zombie Aqua viability dye (BioLegend, 1:400 dilution). After washing with 2% FBS in DPBS, cells were fixed with 4% paraformaldehyde (Santa Cruz Biotechnology) at room temperature for 15 min. Cells were washed again with 2% FBS in DPBS and permeabilized with 100μL of 1× Click-iT saponin-based permeabilization (Thermo Fisher Scientific) at room temperature for 15 min. Cells were stained with P16 INK4A antibody (Cell Signaling Technology, 1:100 dilution) at 4°C overnight. After washing with 2% FBS in DPBS, samples were stained with Alexa Fluor 488- labeled secondary antibodies (Thermo Fisher Scientific, 1:1,000 dilution) (Alexa Fluor 488 or Alexa Fluor 647; Thermo Fisher Scientific) for 45 min at room temperature. After washing with 2% FBS in DPBS, sample acquisition was performed with FACSCanto II (BD Biosciences). Data were analyzed using the FlowJo software.

Lipofuscin analysis

Lipofuscin analysis via flow cytometry was performed using GLF16 (QR LABS), as recently described.[55] Briefly, 1×10^5 cells were washed with 2% FBS in DPBS and stained for 15 min at 4°C with anti-human CD34 BV421 (BioLegend, 1:100 dilution) and Zombie Aqua viability dye (BioLegend, 1:400 dilution). After washing with 2% FBS in DPBS, cells were fixed with 4% paraformaldehyde (Santa Cruz Biotechnology) at room temperature for 15 min. Cells were washed again with 2% FBS in DPBS and permeabilized with 500 μL of 0.3% Triton X-100 (Sigma-Aldrich) at room temperature for 20 min. After centrifugation and removal of excess permeabilization solution, cells were stained with 50 μL of GLF solution (1:40 dilution of 200 μg/mL GLF stock solution in 2.5% DMSO + 2.5% TWEEN 20 (Sigma-Aldrich)) at room temperature for 8 min. After washing once with 2.5% DMSO + 2.5% TWEEN 20 in DPBS and once with 2% FBS in DPBS, sample acquisition was performed with FACSCanto II (BD Biosciences). Data were analyzed using the FlowJo software.

Mitochondrial mass analysis

Mitochondrial mass analysis was performed using the MitoTracker Green FM Dye (Thermo Fisher Scientific). Briefly, 1×10^5 cells were washed with 2% FBS in DPBS and stained for 15 min at 4°C with anti-human CD34 PE (Miltenyi Biotec, 1:100 dilution) or anti-human CD34 BV421 (BioLegend, 1:100 dilution), anti-human CD90 APC (BD Biosciences, 1:100 dilution), and Zombie Aqua viability dye (BioLegend, 1:400 dilution). After washing in DPBS, cells were resuspended in 1 mL of DPBS and incubated with 1 μL of MitoTracker dye for 30 min in the incubator at 37°C. After washing in 2% FBS in DPBS, samples were immediately acquired with FACSCanto II (BD Biosciences). Data were analyzed using the FlowJo software.

Mice

All experiments and procedures involving animals were performed with the approval of the Animal Care and Use Committee and Ethical Board of the San Raffaele Hospital (IACUC 923, 1183, 1207) and were authorized by the Italian Ministry of Health and local authorities in compliance with Italian law. NOD-scid-Il2rg−/− (NSG) female mice (The Jackson Laboratory) were held under specific-pathogen-free conditions.

CD34$^+$ HSPC xenotransplantation experiments in NSG mice

For xenotransplantation of mPB HSPCs, the outgrowth of 2×10^5 to 5×10^5 HSPCs at the start of the culture (day-0 equivalent) were injected intravenously into sublethally irradiated (180–200 cGy) female NSG mice. Matched numbers of HSPCs were seeded at day 0 of culture for each experimental group to transplant the same number of culture-initiating HSPCs in each mouse. The precise dose of injected cells differed according to the experimental conditions and is described in the figure legend for each experiment. Mice were randomly distributed to each experimental group. Human CD45$^+$ cell engraftment and the presence of transduced cells were monitored by serial collection of peripheral blood (approximately every 4 weeks) and, at the end of the experiment (12–18 weeks after transplantation), BM was collected for endpoint analyses. Hindlimb BM was flushed with PBS 2% FBS and 2×10^6 cells were stained for surface markers. The remaining cells, if required, were used to perform secondary transplantation in NSG mice after bead purification with mouse cell depletion or enriched for human CD34$^+$ cell kit (Miltenyi Biotec) according to the manufacturer's instructions.

Peripheral blood analysis

Mice were bled via the tail vein following analgesia. For each mouse, 250 μL of peripheral blood was added to 10 μL of PBS containing 45-mg/mL EDTA. For immunostaining, a known volume of whole blood (100 μL) was incubated with anti-human Fc receptor-blocking antibodies for 10 min at room temperature and then incubated in the presence of monoclonal antibodies (for a list of antibodies, see Table 1) for 15 min at room temperature. Erythrocytes were removed

by lysis with the TQ-Prep workstation (Beckman Coulter) in the presence of an equal volume of FBS (100 μL).

Bone marrow analysis

At the experimental endpoint, mice were humanely euthanized, and BM cells were obtained by flushing the femurs in PBS 2% FBS solution. Cells (1–2 × 10^6 cells) were washed, resuspended in 100 μL of PBS containing 2% FBS, and incubated with anti-human and/or anti-mouse FcγIII/II receptor (Cd16/Cd32)-blocking antibodies for 15 min at 4°C. Staining was performed with monoclonal antibodies (Table 1) for 20 min at 4°C.

Lentiviral barcode next-generation sequencing library preparation

PCR products compatible with Illumina sequencing were generated by targeted amplification of the integrated LV vector barcode from gDNA isolated from the BM of murine xenografts. A total of 50–100 ng of gDNA was used as input. Amplification was performed using Phusion High-Fidelity DNA Polymerase (Thermo Fisher Scientific) according to the manufacturer's protocol, with the following thermal cycling conditions: initial denaturation at 98°C for 60 s, 26 cycles of denaturation at 98°C for 10 s, annealing at 56°C for 30 s, and extension at 72°C for 15 s, followed by a final extension at 72°C for 10 min.

Custom next-generation sequencing (NGS)-grade primers were used at a final concentration of 10 μM. Primers included Illumina adapter sequences (P5 and read 1 for forward primers; P7 and read 2 for reverse primers) along with unique 8-base sample-specific barcodes (denoted as "xxxxxxxx") for multiplexing.

Primer sequences were as follows:

- Forward primer: 5′-AATGATACGGCGACCACCGAGATCTACACTCTTTCCCTACACGACGCTCTTCCGATCTxxxxxxxxCTACTCAGACAATGCGATGC-3′
- Reverse primer: 5′-CAAGCAGAAGACGGCATACGAGATGTGACTGGAGTTCAGACGTGTGCTCTTCCGATCTxxxxxxxxCGTGCCTTCCTTGACCCTG-3′

PCR products were resolved on a 1.35% agarose gel, and the 255-bp band corresponding to the expected barcode amplicon was excised and purified using the Promega Wizard SV Gel and PCR Clean-Up System in accordance with the manufacturer's instructions. Purified libraries were pooled at equimolar concentrations and subjected to paired-end sequencing (2 × 75 bp) on an Illumina NextSeq platform.

Lentiviral integration-site library preparation

Integration sites were retrieved by Sonication Linker Mediated (SLiM)-PCR, as previously described,[56] with minor modifications. Briefly, for each sample, up to 300 ng of gDNA were processed by DNA shearing using the Covaris E220 Ultrasonicator, generating fragments with an average size of 1,000 bp. The fragmented DNA samples were subjected to end repair and 3′ adenylation and then ligated to linker cassettes containing an 8-nucleotide sequence barcode used for sample identification and all the sequences required for the read 2 paired-end sequencing. The ligation products were split in three technical replicates and subjected to 35 cycles of exponential PCR using primers specific for the LV vector long terminal repeats (LTR) and the linker cassette. A subsequent amplification with additional 10 PCR cycles was performed using a primer specific for the linker cassette and the LTR. These primers contain an 8-nucleotide length barcode used for sample identification (coupled with the barcode on the linker cassette) and the sequences needed for the read 1 sequencing, plus a sequence of 12 random nucleotides allowing easier cluster recognition in the first sequencing cycles on the NGS sequencer. The generated SLiM-PCR products are thus associated to a unique pair of barcodes, assembled into libraries, and subjected to NGS Illumina sequencing.

LVIS bioinformatic analysis

Sequencing reads were processed using VISPA2[57] that isolates genomic sequences flanking the vector LTR and maps them to the human genome (hg19). Because vector integration in the same genomic position in different cells is a very low probability event, identical integration sites (IS) in independent samples were considered as contamination or amplification artifacts, which may occur during the technical procedure. Datasets were pruned from potential contaminations and false positives between each primary mouse and from IS deriving from unrelated secondary mice. IS with identical genomic coordinates shared between mice belonging to different experimental groups were reassigned based on identification of the integration site by at least two SLiM technical replicates and sequence-count numbers. Downstream analyses of vector integration sites, such as relative abundance analysis, sharing analysis and common integration site (CIS) analysis, were performed using ISAnalytics.[58] Clonal abundance estimates as the relative percentage of genome numbers was determined by the R package "sonicLength."[59] Common insertion sites were calculated by the Grubbs test for outliers.[60]

Lentiviral barcoding preprocessing and quantification

Demultiplexed raw data were quality checked using fastqc tool to check for low-quality samples in terms of base quality scores and to check the presence of a barcoded region characterized by high heterogeneity. All samples were down sampled to 300k input reads using seqtk (1.4-r130) to avoid coverage biases and allow direct comparison of all runs and conditions, as well ensure reproducibility using a fixed seed. The distribution of barcodes counts and Shannon entropy indexes were compared between raw and subsampled samples to verify that complexity and library saturation were maintained.

Identification and quantification of barcodes was performed with barseq[61] setting edit distance and minimal counts to 2 and 3 respectively. Barcode extraction parametrization was the following: -1 P:CTACTCAGACAATGCGATGC -2 S:AATTTCCTCATTTTATT -3 R:N -4 S:TACGTCGA -5 P:GCGAGGA. Extraction metrics

provided by TagDust v2.33 were hence inspected to identify and discard samples with low extraction rates (<80%).

Lentiviral barcoding processing and downstream analysis

To assess the threshold for the minimum barcode abundance to identify a clone, we employed a strategy that relies on the comparison of shared clones between 1^{ary} and 2^{ary} transplant samples. We leveraged SCGM(CG) barcoded libraries treated with UM171 (UM) and UM171+SR1 (US) for which primary and secondary transplant data were available (Data S4). Using raw barcode counts provided by barseq, we looked for the lowest frequency barcode in first transplant that was expanded in second transplant. Barcodes present in more than three mice in first transplant group were considered artifacts and discarded. The identified threshold equal to (0.005%) was then applied to all the experimental groups analyzed in this work. Barcodes with frequency less than 0.005% were considered artifacts, contaminants, or unreliable.

Moreover, we applied two strategies to further discard or minimize contaminants. The first strategy aimed to discard barcodes that were shared in more than n animals (Nmax parameter, see code for details) within each experimental group, whereas the second one consisted in the re-assignment of barcodes that were shared between independent groups. In particular, the barcode was reassigned to the top sample whose quantification was 10× higher than the second most abundant sample sharing the barcode. If none of the samples met this rule, the barcode was considered shared across samples of independent groups.

We focused on the evaluation of the barcodes sharing within and between groups of samples as well as the clonal distribution and complexity. This latter topic was assessed evaluating the Shannon entropy index using the vegan R package (v.2.5-7).

Barcode count data processing and figure preparation was performed within R environment (v4.1.0), using several packages including among the most used: ggplot2 (v3.3.5), ggalluvial (v0.12.3), and dplyr (v1.0.8).

Bulk RNA sequencing

Raw data in fastq format were quality control (QC)-checked and trimmed using TrimGalore (v0.5.0) to get rid of adapters at 3′ of reads. Trimmed reads were then aligned to the GRCh38 reference genome using STAR (v2.7.0d). Gencode genes primary assembly gene-transfer file (GTF) v35 was used as reference gene annotation file. Post-alignment metrics, including coverage distribution across gene length and percentage of reads mapping to exons, were collected by using Qorts (v1.3.5). We then assigned reads to genes (gene counting) by using feature counts (v1.6.3). Gene-count matrices were analyzed in the R environment (v4.0.3). We employed a data-analysis workflow that relied on edgeR identification of differentially expressed genes (DEGs) setting custom biological variation coefficients (BCVs) to 0.1–0.3 for the different 1-vs-1 comparisons performed (72 vs. 24 h in each batch separately). Counts were normalized using the Trimmed Mean of M-values (TMM) method, and the differential test was performed with the exactTest function provided by the edgeR package (v3.32). Genes with an adjusted $p < 0.05$ (Benjamini-Hochberg false discovery rate [FDR] method) were considered differentially expressed.

DEG lists and pre-ranked gene lists according to log2FC were used for performing over-representation analysis (ORA) and gene set enrichment analysis (GSEA), respectively. Terms with an adjusted $p < 0.05$ were considered statistically significant. Analyses and charts were produced with clusterProfiler (v4.7.1),[62] enrichplot (v1.16.2), and ggplot2 (v3.3.5) R packages.

GSEA pre-ranked list for the evaluation of the transduction timing was prepared calculating a mean fold change value of the two batches adding an offset of 1 to avoid infinite values in log transformation. Both ORA and GSEA were computed considering different reference datasets including Gene Ontology, Kyoto Encyclopedia of Genes and Genomes (KEGG) pathway database, Reactome pathway database, and Molecular Signatures Database (MSigDB). In addition, for the evaluation of senescence signatures, we relied on a custom gmt reference file that includes literature and database-based signatures.

scRNA-seq

HSPCs were resuspended at the appropriate concentration for loading into the Chromium 10X single-cell 3′ Gene Expression v2 or v3 chip according to the manufacturer's protocol. 3′ gene expression libraries construction and sequencing on Illumina platforms NextSeq or NovaSeq S1 were performed following the manufacturer's indications.

Raw data were processed by Cell Ranger Single-Cell Software Suite, with cellranger count pipeline (version 7.0.1, 10X Genomics) to produce feature-barcodes matrices with Unique Molecular Identifier (UMI) counts for each cell. GRCh38 genome genes annotations provided by the manufacturer were used as reference data. Feature barcode counts matrices were then imported and processed within the R environment (v4.1.3) and analyzed with several R and Bioconductor packages. Figures 2C and 2H datasets were analyzed with Seurat R package.[63] Figure 2H dataset raw matrix was pre-processed with the adaptively thresholded low-rank approximation (ALRA) algorithm using default settings. For both datasets, we performed quality control by discarding cells with lower number of expressed genes and genes expressed by few cells. Counts were log normalized with a scale factor of 10,000, and most variable genes were identified (the top 15% of expressed genes). Normalized data were then scaled accounting for cell cycle (CC.Difference), total depth (nCount_RNA), and mitochondrial content (pct_mito). Hence, we performed dimensionality reduction by calculating principal components (PCs) and selecting the top components based on the elbow rule. Harmony methods were employed to remove potential batch effects, using sample identity or time point + patient (pt.tp) as the batch variable for Figures 2C and 2H datasets, respectively. Clustering was computed by Louvain improved algorithm on neighborhood graph built on top harmony

components. Marker genes for each cluster were computed using the FindAllMarker function in Seurat package. Parameters used for each dataset can be found in the GitLab repository provided with the manuscript. For Figure 2C dataset, cluster annotation was manually curated by inspecting marker genes and SingleR (v1.8.1) classification,[64] using the dataset from Sakurai et al. as reference.[23] As a result, we obtained a two-layer annotation, a more granular one referred to as classification variable and a less granular one named population to ease data interpretation. For the Figure 2H dataset, cell annotation was performed with scGATE (v1.6.2) R package[65] using the Figure 2C dataset as reference.

To assess the effect of expansion culture on mPB CD34$^+$ cells, we identified genes significantly deregulated across culture time points starting from uncultured (day 0) cells to day-4 and day-8 expansion time points. To correct for differences in cell population abundances, we performed all comparisons across time points within each cell population (classification label). First, we identified differentially expressed genes between time points (day 4 vs. day 0; day 8 vs. day 4, and day 8 vs. day 0) by the FindMarker function from Seurat R package. According to fold-change direction, we defined different patterns of modulation that could be simplified as up- or downregulated across culture (consistently up/down, early or late up/down according to day-4 and day-8 significance and direction). We next looked for a more comprehensive set of genes that could summarize a global culture effect across most populations, and selected sets of common up- and downregulated genes shared in at least four populations out of seven identified by our population label. Lists of common up-/downregulated genes across culture were used as input for ORA using clusterprofiler R package (v4.7.1).[62] Enrichment terms (adjusted $p < 0.05$; Benjamini-Hochberg correction) were then plotted with the cnetplot function provided in the enrichplot r package (v1.14.2). To assess senescence and DNA Damage Response (DDR)-associated transcriptional programs across culture time points, we quantified expression of several gene panels within our datasets by UCell's rank-based gene signature scoring (AddModuleScore_UCell). Per-cell UCell scores were then aggregated as median values within HSC cluster (Figure 2C) and bulk (non-HSC clusters) compartments at each time point.

To evaluate the transduction efficiency of MPS patient samples, we assessed for expression of WPRE (LV-specific gene) transcripts as surrogate of the IDUA LV-derived transgene, which itself could not be discriminated by IDUA transcripts originating from the native gene. Alignment and quantification of WPRE transcripts was ensured by adding the WPRE sequence to the reference human genome in cellranger (10X Genomics). Transduction efficiency (TE) was calculated as the fraction of cells bearing the WPRE transcripts within each sample and/or cell population.

Statistical analysis

The number of biologically independent samples, animals, or experiments is indicated by *n*. For some experiments, different HSPC donors were pooled to account for donor-related variability and to achieve a sufficient number of cells required for the experiment. In all studies, values are expressed as means ± SEM unless otherwise indicated.

Two-tailed tests were performed throughout the study. The Mann-Whitney test was performed to compare two independent groups, while, in the presence of more than two independent groups, the Kruskal-Wallis test followed by *post hoc* analysis using Dunn's test was used. In all box plots shown in the figure, the whiskers represent the minimum and maximum values of the data. Throughout the manuscript significance levels are coded as follows: $^{*}p < 0.05$, $^{**}p < 0.01$, $^{***}p < 0.001$. For the cytokine-optimization experiments (Figures 3A–3C), linear mixed-effects models were fitted using the lme4 and lmerTest R packages, with IL-3 dose and IL-6 dose as fixed effects (including their interaction) and donor as a random intercept. Overall effects were assessed by type III ANOVA with Satterthwaite's approximation for denominator degrees of freedom. *Post hoc* pairwise comparisons of estimated marginal means (emmeans package) were adjusted using the Holm method (Table S8). ELDA was performed by accessing https://bioinf.wehi.edu.au/software/elda/.[66]

Statistical testing, if not specified otherwise, was performed with GraphPad Prism software (v. 10.6.1) or in the R environment (R v. 4.1.0).

DATA AND CODE AVAILABILITY

- Lentiviral barcoding and integration site analysis raw data were deposited on ENA (ENA: PRJEB76976, PRJEB77101, PRJEB77102, and PRJEB94025). Bulk RNA sequencing raw data were deposited at the GEO repository (GEO: GSE292605). scRNA-seq raw data were deposited under GSE292604 and GSE292606 GEO repositories for Figures 2H and 2C datasets, respectively. Code used to analyze data can be found in the following GitLab repository: http://www.bioinfotiget.it/gitlab/custom/zonari_mpbhscexp_2025.
- Materials can be made available upon signing a material transfer agreement with standard provisions, except UM171 (requests to be made to ExCellThera) and patient material/data from the MPS1-H study (requests to be approved by Orchard Therapeutics Ltd. (OTL).

ACKNOWLEDGMENTS

The authors thank members of the Gentner lab (former lab in Milan and present lab in Lausanne) for help with experiments, discussion and insight; the Fractal facility personnel for cell sorting; the Center for Omics Sciences (COSR) for advice and assistance with genomic sequencing; Cristina Tresoldi and the OSR biobank for sample collection and storage; the Pediatric and Bone Marrow Transplant Unit personnel, in particular Maria Ester Bernardo and Alessandro Aiuti, for helpful discussions and advise on the MPSI-H trial; and co-investigators from the XPAND consortium, in particular Luigi Naldini and Samuele Ferrari, for helpful discussions. We furthermore thank Adam Wilkinson for helpful discussions on the ChemD medium. We thank Fabrizio Benedicenti for his support in designing the LV barcode amplification strategy. This work was supported by grants to B.G. from Fondazione Telethon ETS (SR-TIGET core grant 2016 and 2021, ref. C1/3309), the European Union Horizon Europe program under grant agreement no. 101070950 (X-PAND) and the European Union - Next Generation EU, Mission 4, Component 2, CUP G83C22000270001, and a grant to E.Z. from the Italian Ministry of Health (Ministero della Salute, grant GR-2019-12369499).

AUTHOR CONTRIBUTIONS

Conceptualization, E.Z., M.M.N., M.B., and B.G.; methodology, E.Z., M.M.N., M.B., M. Volpin, S.Z.X., J.E.D., and B.G.; formal analysis, E.Z., M.M.N., M.B., M. Volpin, and B.G.; investigation, E.Z., M.M.N., M.B., M. Volpin, F.V., G.D., C.C., I.G., B.M., L.O., I.V., M. Vezzoli, L.H., F.M., G.F., and R.V.; resources, M. Volpin, F.T., L.O., D.L.,

I.M., E.M., and B.G.; data curation, M.B.; visualization, E.Z., M.M.N., M.B., and B.G.; funding acquisition, E.Z. and B.G.; project administration, E.Z., M.M.N., M.B., G.D., and B.G.; supervision, R.D.M., E.M., and B.G.; writing – original draft, E.Z., M.M.N., M.B., and B.G.; writing – review and editing, E.Z., M.M.N., M.B., M. Volpin, S.Z.X., J.E.D., E.M., and B.G.

DECLARATION OF INTERESTS

B.G. participated in the Scientific Advisory Board of ExCellThera from 2021 to 2023. Lentiviral vector-based gene therapy for patients with MPSI-H was licensed to OTL in 2019. OTL and ExCellThera had the opportunity to review the manuscript.

SUPPLEMENTAL INFORMATION

Supplemental information can be found online at https://doi.org/10.1016/j.ymthe.2026.05.014.

REFERENCES

1. Calabria, A., Spinozzi, G., Cesana, D., Buscaroli, E., Benedicenti, F., Pais, G., Gazzo, F., Scala, S., Lidonnici, M.R., Scaramuzza, S., et al. (2024). Long-term lineage commitment in haematopoietic stem cell gene therapy. Nature *636*, 162–171. https://doi.org/10.1038/s41586-024-08250-x.
2. Spencer Chapman, M., Wilk, C.M., Boettcher, S., Mitchell, E., Dawson, K., Williams, N., Müller, J., Kovtonyuk, L., Jung, H., Caiado, F., et al. (2024). Clonal dynamics after allogeneic haematopoietic cell transplantation. Nature *635*, 926–934. https://doi.org/10.1038/s41586-024-08128-y.
3. Hofmann, J., and Kokkaliaris, K.D. (2024). Bone marrow niches for hematopoietic stem cells: life span dynamics and adaptation to acute stress. Blood *144*, 21–34. https://doi.org/10.1182/blood.2023023788.
4. Granot, N., and Storb, R. (2020). History of hematopoietic cell transplantation: challenges and progress. haematol. *105*, 2716–2729. https://doi.org/10.3324/haematol.2019.245688.
5. Delaney, C., Heimfeld, S., Brashem-Stein, C., Voorhies, H., Manger, R.L., and Bernstein, I.D. (2010). Notch-mediated expansion of human cord blood progenitor cells capable of rapid myeloid reconstitution. Nat. Med. *16*, 232–236. https://doi.org/10.1038/nm.2080.
6. Horwitz, M.E., Stiff, P.J., Cutler, C., Brunstein, C., Hanna, R., Maziarz, R.T., Rezvani, A.R., Karris, N.A., McGuirk, J., Valcarcel, D., et al. (2021). Omidubicel vs standard myeloablative umbilical cord blood transplantation: results of a phase 3 randomized study. Blood *138*, 1429–1440. https://doi.org/10.1182/blood.2021011719.
7. Cohen, S., Roy, J., Lachance, S., Delisle, J.-S., Marinier, A., Busque, L., Roy, D.-C., Barabé, F., Ahmad, I., Bambace, N., et al. (2020). Hematopoietic stem cell transplantation using single UM171-expanded cord blood: a single-arm, phase 1-2 safety and feasibility study. Lancet Haematol. *7*, e134–e145. https://doi.org/10.1016/S2352-3026(19)30202-9.
8. Wagner, J.E., Brunstein, C.G., Boitano, A.E., DeFor, T.E., McKenna, D., Sumstad, D., Blazar, B.R., Tolar, J., Le, C., Jones, J., et al. (2016). Phase I/II Trial of StemRegenin-1 Expanded Umbilical Cord Blood Hematopoietic Stem Cells Supports Testing as a Stand-Alone Graft. Cell Stem Cell *18*, 144–155. https://doi.org/10.1016/j.stem.2015.10.004.
9. Signer, R.A.J., Magee, J.A., Salic, A., and Morrison, S.J. (2014). Haematopoietic stem cells require a highly regulated protein synthesis rate. Nature *509*, 49–54. https://doi.org/10.1038/nature13035.
10. Huang, J., Nguyen-McCarty, M., Hexner, E.O., Danet-Desnoyers, G., and Klein, P.S. (2012). Maintenance of hematopoietic stem cells through regulation of Wnt and mTOR pathways. Nat. Med. *18*, 1778–1785. https://doi.org/10.1038/nm.2984.
11. Johnson, C.S., Williams, M., Sham, K., Belluschi, S., Ma, W., Wang, X., Lau, W.W.Y., Kaufmann, K.B., Krivdova, G., Calderbank, E.F., et al. (2024). Adaptation to ex vivo culture reduces human hematopoietic stem cell activity independently of the cell cycle. Blood *144*, 729–741. https://doi.org/10.1182/blood.2023021426.
12. Fares, I., Chagraoui, J., Gareau, Y., Gingras, S., Ruel, R., Mayotte, N., Csaszar, E., Knapp, D.J.H.F., Miller, P., Ngom, M., et al. (2014). Pyrimidoindole derivatives are agonists of human hematopoietic stem cell self-renewal. Science *345*, 1509–1512. https://doi.org/10.1126/science.1256337.
13. Chagraoui, J., Girard, S., Spinella, J.-F., Simon, L., Bonneil, E., Mayotte, N., MacRae, T., Coulombe-Huntington, J., Bertomeu, T., Moison, C., et al. (2021). UM171 Preserves Epigenetic Marks that Are Reduced in Ex Vivo Culture of Human HSCs via Potentiation of the CLR3-KBTBD4 Complex. Cell Stem Cell *28*, 48–62.e6. https://doi.org/10.1016/j.stem.2020.12.002.
14. Chaurasia, P., Gajzer, D.C., Schaniel, C., D'Souza, S., and Hoffman, R. (2014). Epigenetic reprogramming induces the expansion of cord blood stem cells. J. Clin. Invest. *124*, 2378–2395. https://doi.org/10.1172/JCI70313.
15. Hua, P., Hester, J., Adigbli, G., Li, R., Psaila, B., Roy, A., Bataille, C.J.R., Wynne, G.M., Jackson, T., Milne, T.A., et al. (2020). The BET inhibitor CPI203 promotes ex vivo expansion of cord blood long-term repopulating HSCs and megakaryocytes. Blood *136*, 2410–2415. https://doi.org/10.1182/blood.2020005357.
16. Horwitz, M.E., Chao, N.J., Rizzieri, D.A., Long, G.D., Sullivan, K.M., Gasparetto, C., Chute, J.P., Morris, A., McDonald, C., Waters-Pick, B., et al. (2014). Umbilical cord blood expansion with nicotinamide provides long-term multilineage engraftment. J. Clin. Invest. *124*, 3121–3128. https://doi.org/10.1172/JCI74556.
17. Rentas, S., Holzapfel, N.T., Belew, M.S., Pratt, G.A., Voisin, V., Wilhelm, B.T., Bader, G.D., Yeo, G.W., and Hope, K.J. (2016). Musashi-2 attenuates AHR signalling to expand human haematopoietic stem cells. Nature *532*, 508–511. https://doi.org/10.1038/nature17665.
18. Boitano, A.E., Wang, J., Romeo, R., Bouchez, L.C., Parker, A.E., Sutton, S.E., Walker, J.R., Flaveny, C.A., Perdew, G.H., Denison, M.S., et al. (2010). Aryl hydrocarbon receptor antagonists promote the expansion of human hematopoietic stem cells. Science *329*, 1345–1348. https://doi.org/10.1126/science.1191536.
19. Guo, B., Huang, X., Lee, M.R., Lee, S.A., and Broxmeyer, H.E. (2018). Antagonism of PPAR-γ signaling expands human hematopoietic stem and progenitor cells by enhancing glycolysis. Nat. Med. *24*, 360–367. https://doi.org/10.1038/nm.4477.
20. Vannini, N., Campos, V., Girotra, M., Trachsel, V., Rojas-Sutterlin, S., Tratwal, J., Ragusa, S., Stefanidis, E., Ryu, D., Rainer, P.Y., et al. (2019). The NAD-Booster Nicotinamide Riboside Potently Stimulates Hematopoiesis through Increased Mitochondrial Clearance. Cell Stem Cell *24*, 405–418.e7. https://doi.org/10.1016/j.stem.2019.02.012.
21. Xie, S.Z., Garcia-Prat, L., Voisin, V., Ferrari, R., Gan, O.I., Wagenblast, E., Kaufmann, K.B., Zeng, A.G.X., Takayanagi, S.-I., Patel, I., et al. (2019). Sphingolipid Modulation Activates Proteostasis Programs to Govern Human Hematopoietic Stem Cell Self-Renewal. Cell Stem Cell *25*, 639–653.e7. https://doi.org/10.1016/j.stem.2019.09.008.
22. Itkin, T., Houghton, S., Schreiner, R., Lin, Y., Badwe, C.R., Voisin, V., Murison, A., Seyedhassantehrani, N., Kaufmann, K.B., Garcia-Prat, L., et al. (2025). Transcriptional activation of regenerative hematopoiesis via microenvironmental sensing. Nat. Immunol. *26*, 378–390. https://doi.org/10.1038/s41590-025-02087-w.
23. Sakurai, M., Ishitsuka, K., Ito, R., Wilkinson, A.C., Kimura, T., Mizutani, E., Nishikii, H., Sudo, K., Becker, H.J., Takemoto, H., et al. (2023). Chemically defined cytokine-free expansion of human haematopoietic stem cells. Nature *615*, 127–133. https://doi.org/10.1038/s41586-023-05739-9.
24. Bai, T., Li, J., Sinclair, A., Imren, S., Merriam, F., Sun, F., O'Kelly, M.B., Nourigat, C., Jain, P., Delrow, J.J., et al. (2019). Expansion of primitive human hematopoietic stem cells by culture in a zwitterionic hydrogel. Nat. Med. *25*, 1566–1575. https://doi.org/10.1038/s41591-019-0601-5.
25. Bruschi, M., Vanzolini, T., Sahu, N., Balduini, A., Magnani, M., and Fraternale, A. (2022). Functionalized 3D scaffolds for engineering the hematopoietic niche. Front. Bioeng. Biotechnol. *10*, 968086. https://doi.org/10.3389/fbioe.2022.968086.
26. Chatterjee, C., Schertl, P., Frommer, M., Ludwig-Husemann, A., Mohra, A., Dilger, N., Naolou, T., Meermeyer, S., Bergmann, T.C., Alonso Calleja, A., and Lee-Thedieck, C. (2021). Rebuilding the hematopoietic stem cell niche: Recent developments and future prospects. Acta Biomater. *132*, 129–148. https://doi.org/10.1016/j.actbio.2021.03.061.
27. Ferrari, G., Thrasher, A.J., and Aiuti, A. (2021). Gene therapy using haematopoietic stem and progenitor cells. Nat. Rev. Genet. *22*, 216–234. https://doi.org/10.1038/s41576-020-00298-5.
28. Ferrari, S., Valeri, E., Conti, A., Scala, S., Aprile, A., Di Micco, R., Kajaste-Rudnitski, A., Montini, E., Ferrari, G., Aiuti, A., and Naldini, L. (2023). Genetic engineering

meets hematopoietic stem cell biology for next-generation gene therapy. Cell Stem Cell *30*, 549–570. https://doi.org/10.1016/j.stem.2023.04.014.

29. Scala, S., Ferrua, F., Basso-Ricci, L., Dionisio, F., Omrani, M., Quaranta, P., Jofra Hernandez, R., Del Core, L., Benedicenti, F., Monti, I., et al. (2023). Hematopoietic reconstitution dynamics of mobilized- and bone marrow-derived human hematopoietic stem cells after gene therapy. Nat. Commun. *14*, 3068. https://doi.org/10.1038/s41467-023-38448-y.

30. Gentner, B., Tucci, F., Galimberti, S., Fumagalli, F., De Pellegrin, M., Silvani, P., Camesasca, C., Pontesilli, S., Darin, S., Ciotti, F., et al. (2021). Hematopoietic Stem- and Progenitor-Cell Gene Therapy for Hurler Syndrome. N. Engl. J. Med. Overseas. Ed. *385*, 1929–1940. https://doi.org/10.1056/NEJMoa2106596.

31. Zonari, E., Desantis, G., Petrillo, C., Boccalatte, F.E., Lidonnici, M.R., Kajaste-Rudnitski, A., Aiuti, A., Ferrari, G., Naldini, L., and Gentner, B. (2017). Efficient Ex Vivo Engineering and Expansion of Highly Purified Human Hematopoietic Stem and Progenitor Cell Populations for Gene Therapy. Stem Cell Rep. *8*, 977–990. https://doi.org/10.1016/j.stemcr.2017.02.010.

32. Zimran, E., Papa, L., Djedaini, M., Patel, A., Iancu-Rubin, C., and Hoffman, R. (2020). Expansion and preservation of the functional activity of adult hematopoietic stem cells cultured ex vivo with a histone deacetylase inhibitor. Stem Cells Transl. Med. *9*, 531–542. https://doi.org/10.1002/sctm.19-0199.

33. Piras, F., Riba, M., Petrillo, C., Lazarevic, D., Cuccovillo, I., Bartolaccini, S., Stupka, E., Gentner, B., Cittaro, D., Naldini, L., and Kajaste-Rudnitski, A. (2017). Lentiviral vectors escape innate sensing but trigger p53 in human hematopoietic stem and progenitor cells. EMBO Mol. Med. *9*, 1198–1211. https://doi.org/10.15252/emmm.201707922.

34. Capo, V., Penna, S., Merelli, I., Barcella, M., Scala, S., Basso-Ricci, L., Draghici, E., Palagano, E., Zonari, E., Desantis, G., et al. (2020). Expanded circulating hematopoietic stem/progenitor cells as novel cell source for the treatment of TCIRG1 osteopetrosis. haematol. *106*, 74–86. https://doi.org/10.3324/haematol.2019.238261.

35. Wang, J.C.Y., Doedens, M., and Dick, J.E. (1997). Primitive human hematopoietic cells are enriched in cord blood compared with adult bone marrow or mobilized peripheral blood as measured by the quantitative in vivo SCID-repopulating cell assay. Blood *89*, 3919–3924.

36. Hammond, C.A., Wu, S.W., Wang, F., MacAldaz, M.E., and Eaves, C.J. (2023). Aging alters the cell cycle control and mitogenic signaling responses of human hematopoietic stem cells. Blood *141*, 1990–2002. https://doi.org/10.1182/blood.2022017174.

37. Pievani, A., Granata, V., Desantis, G., Antolini, L., Ornaghi, S., Galleu, A., Biondi, A., Gentner, B., Dazzi, F., and Serafini, M. (2022). CD14 positive cells accelerate hematopoietic stem cell engraftment. Bone Marrow Transpl. *57*, 942–948. https://doi.org/10.1038/s41409-022-01662-1.

38. Quaranta, P., Basso-Ricci, L., Jofra Hernandez, R., Pacini, G., Naldini, M.M., Barcella, M., Seffin, L., Pais, G., Spinozzi, G., Benedicenti, F., et al. (2024). Circulating hematopoietic stem/progenitor cell subsets contribute to human hematopoietic homeostasis. Blood *143*, 1937–1952. https://doi.org/10.1182/blood.2023022666.

39. Wu, X., Dao Thi, V.L., Huang, Y., Billerbeck, E., Saha, D., Hoffmann, H.-H., Wang, Y., Silva, L.A.V., Sarbanes, S., Sun, T., et al. (2018). Intrinsic Immunity Shapes Viral Resistance of Stem Cells. Cell *172*, 423–438.e25. https://doi.org/10.1016/j.cell.2017.11.018.

40. della Volpe, L., Midena, F., Vacca, R., Tavella, T., Alessandrini, L., Farina, G., Brandas, C., Lo Furno, E., Giannetti, K., Carsana, E., et al. (2024). A p38 MAPK-ROS axis fuels proliferation stress and DNA damage during CRISPR-Cas9 gene editing in hematopoietic stem and progenitor cells. Cell Rep. Med. *5*, 101823. https://doi.org/10.1016/j.xcrm.2024.101823.

41. Notta, F., Doulatov, S., Laurenti, E., Poeppl, A., Jurisica, I., and Dick, J.E. (2011). Isolation of single human hematopoietic stem cells capable of long-term multilineage engraftment. Science *333*, 218–221. https://doi.org/10.1126/science.1201219.

42. Anjos-Afonso, F., Buettner, F., Mian, S.A., Rhys, H., Perez-Lloret, J., Garcia-Albornoz, M., Rastogi, N., Ariza-McNaughton, L., and Bonnet, D. (2022). Single cell analyses identify a highly regenerative and homogenous human CD34+ hematopoietic stem cell population. Nat. Commun. *13*, 2048. https://doi.org/10.1038/s41467-022-29675-w.

43. Coulombe, P., Tomellini, E., Chagraoui, J., Mayotte, N., and Sauvageau, G. (2025). Deciphering the effect of UM171 on human hematopoietic progenitor cell fate through clonal analysis. Nat. Commun. *16*, 195. https://doi.org/10.1038/s41467-024-55225-7.

44. Kaufmann, K.B., Zeng, A.G.X., Coyaud, E., Garcia-Prat, L., Papalexi, E., Murison, A., Laurent, E.M.N., Chan-Seng-Yue, M., Gan, O.I., Pan, K., et al. (2021). A latent subset of human hematopoietic stem cells resists regenerative stress to preserve stemness. Nat. Immunol. *22*, 723–734. https://doi.org/10.1038/s41590-021-00925-1.

45. Lechman, E.R., Gentner, B., van Galen, P., Giustacchini, A., Saini, M., Boccalatte, F.E., Hiramatsu, H., Restuccia, U., Bachi, A., Voisin, V., et al. (2012). Attenuation of miR-126 activity expands HSC in vivo without exhaustion. Cell Stem Cell *11*, 799–811. https://doi.org/10.1016/j.stem.2012.09.001.

46. Laurenti, E., Frelin, C., Xie, S., Ferrari, R., Dunant, C.F., Zandi, S., Neumann, A., Plumb, I., Doulatov, S., Chen, J., et al. (2015). CDK6 levels regulate quiescence exit in human hematopoietic stem cells. Cell Stem Cell *16*, 302–313. https://doi.org/10.1016/j.stem.2015.01.017.

47. Bozhilov, Y., Brown, E., Hsu, I., Singh, I., Rodriguez-Fraticelli, A., Roy, A., Yamazaki, S., and Wilkinson, A.C. (2024). Reducing oxidative stress improves ex vivo polymer-based human haematopoietic stem and progenitor cell culture and gene editing. Preprint at bioRxiv. https://doi.org/10.1101/2024.09.17.613552.

48. Horton, P.D., Dumbali, S., and Wenzel, P.L. (2020). Mechanoregulation in hematopoiesis and hematologic disorders. Curr. Stem Cell Rep. *6*, 86–95. https://doi.org/10.1007/s40778-020-00172-4.

49. Capo, V., Abinun, M., and Villa, A. (2022). Osteoclast rich osteopetrosis due to defects in the TCIRG1 gene. Bone *165*, 116519. https://doi.org/10.1016/j.bone.2022.116519.

50. Biffi, A., Montini, E., Lorioli, L., Cesani, M., Fumagalli, F., Plati, T., Baldoli, C., Martino, S., Calabria, A., Canale, S., et al. (2013). Lentiviral hematopoietic stem cell gene therapy benefits metachromatic leukodystrophy. Science *341*, 1233158. https://doi.org/10.1126/science.1233158.

51. Aiuti, A., Biasco, L., Scaramuzza, S., Ferrua, F., Cicalese, M.P., Baricordi, C., Dionisio, F., Calabria, A., Giannelli, S., Castiello, M.C., et al. (2013). Lentiviral hematopoietic stem cell gene therapy in patients with Wiskott-Aldrich syndrome. Science *341*, 1233151. https://doi.org/10.1126/science.1233151.

52. Follenzi, A., Ailles, L.E., Bakovic, S., Geuna, M., and Naldini, L. (2000). Gene transfer by lentiviral vectors is limited by nuclear translocation and rescued by HIV-1 pol sequences. Nat. Genet. *25*, 217–222. https://doi.org/10.1038/76095.

53. Soldi, M., Sergi, L.S., Unali, G., Kerzel, T., Cuccovillo, I., Capasso, P., Annoni, A., Biffi, M., Rancoita, P.M.V., Cantore, A., et al. (2020). Laboratory-Scale Lentiviral Vector Production and Purification for Enhanced Ex Vivo and In Vivo Genetic Engineering. Mol. Ther. Methods Clin. Dev. *19*, 411–425. https://doi.org/10.1016/j.omtm.2020.10.009.

54. Conti, A., Giannetti, K., Midena, F., Beretta, S., Gualandi, N., De Marco, R., Carsana, E., Varesi, A., Tavella, T., Alessandrini, L., et al. (2025). Senescence and inflammation are unintended adverse consequences of CRISPR-Cas9/AAV6-mediated gene editing in hematopoietic stem cells. Cell Rep. Med. *6*, 102157. https://doi.org/10.1016/j.xcrm.2025.102157.

55. Magkouta, S., Veroutis, D., Pousias, A., Papaspyropoulos, A., Pippa, N., Lougiakis, N., Kambas, K., Lagopati, N., Polyzou, A., Georgiou, M., et al. (2023). A fluorophore-conjugated reagent enabling rapid detection, isolation and live tracking of senescent cells. Mol. Cell *83*, 3558–3573.e7.

56. Cesana, D., Calabria, A., Rudilosso, L., Gallina, P., Benedicenti, F., Spinozzi, G., Schiroli, G., Magnani, A., Acquati, S., Fumagalli, F., et al. (2021). Retrieval of vector integration sites from cell-free DNA. Nat. Med. *27*, 1458–1470. https://doi.org/10.1038/s41591-021-01389-4.

57. Spinozzi, G., Calabria, A., Brasca, S., Beretta, S., Merelli, I., Milanesi, L., and Montini, E. (2017). VISPA2: a scalable pipeline for high-throughput identification and annotation of vector integration sites. BMC Bioinformatics *18*, 520. https://doi.org/10.1186/s12859-017-1937-9.

58. Pais, G., Spinozzi, G., Cesana, D., Benedicenti, F., Albertini, A., Bernardo, M.E., Gentner, B., Montini, E., and Calabria, A. (2023). ISAnalytics enables longitudinal and high-throughput clonal tracking studies in hematopoietic stem cell gene therapy applications. Brief. Bioinform. *24*, bbac551. https://doi.org/10.1093/bib/bbac551.

59. Berry, C.C., Gillet, N.A., Melamed, A., Gormley, N., Bangham, C.R.M., and Bushman, F.D. (2012). Estimating abundances of retroviral insertion sites from DNA fragment length data. Bioinformatics *28*, 755–762. https://doi.org/10.1093/bioinformatics/bts004.

60. Biffi, A., Bartolomae, C.C., Cesana, D., Cartier, N., Aubourg, P., Ranzani, M., Cesani, M., Benedicenti, F., Plati, T., Rubagotti, E., et al. (2011). Lentiviral vector common integration sites in preclinical models and a clinical trial reflect a benign integration bias and not oncogenic selection. Blood *117*, 5332–5339. https://doi.org/10.1182/blood-2010-09-306761.

61. Ferrari, S., Beretta, S., Jacob, A., Cittaro, D., Albano, L., Merelli, I., Naldini, L., and Genovese, P. (2021). BAR-Seq clonal tracking of gene-edited cells. Nat. Protoc. *16*, 2991–3025. https://doi.org/10.1038/s41596-021-00529-x.

62. Wu, T., Hu, E., Xu, S., Chen, M., Guo, P., Dai, Z., Feng, T., Zhou, L., Tang, W., Zhan, L., et al. (2021). clusterProfiler 4.0: A universal enrichment tool for interpreting omics data. The Innovation *2*, 100141. https://doi.org/10.1016/j.xinn.2021.100141.

63. Hao, Y., Hao, S., Andersen-Nissen, E., Mauck, W.M., III, Zheng, S., Butler, A., Lee, M.J., Wilk, A.J., Darby, C., Zager, M., et al. (2021). Integrated analysis of multimodal single-cell data. Cell *184*, 3573–3587.e29. https://doi.org/10.1016/j.cell.2021.04.048.

64. Aran, D., Looney, A.P., Liu, L., Wu, E., Fong, V., Hsu, A., Chak, S., Naikawadi, R.P., Wolters, P.J., Abate, A.R., et al. (2019). Reference-based analysis of lung single-cell sequencing reveals a transitional profibrotic macrophage. Nat. Immunol. *20*, 163–172. https://doi.org/10.1038/s41590-018-0276-y.

65. Andreatta, M., Berenstein, A.J., and Carmona, S.J. (2022). scGate: marker-based purification of cell types from heterogeneous single-cell RNA-seq datasets. Bioinformatics *38*, 2642–2644. https://doi.org/10.1093/bioinformatics/btac141.

66. Hu, Y., and Smyth, G.K. (2009). ELDA: extreme limiting dilution analysis for comparing depleted and enriched populations in stem cell and other assays. J. Immunol. Methods *347*, 70–78. https://doi.org/10.1016/j.jim.2009.06.008.

YMTHE, Volume *34*

Supplemental Information

Ex vivo expansion of hematopoietic stem and progenitor cells from human mobilized peripheral blood for gene therapy applications

Erika Zonari, Matteo Maria Naldini, Matteo Barcella, Monica Volpin, Francesca Vincenti, Giacomo Desantis, Leila Hadadi, Carolina Caserta, Ilaria Galasso, Beatrice Martini, Federico Midena, Giacomo Farina, Roberta Vacca, Francesca Tucci, Leonardo Ormoli, Ilaria Visigalli, Michela Vezzoli, Dejan Lazarevic, Ivan Merelli, Stephanie Z. Xie, John E. Dick, Raffaella Di Micco, Eugenio Montini, and Bernhard Gentner

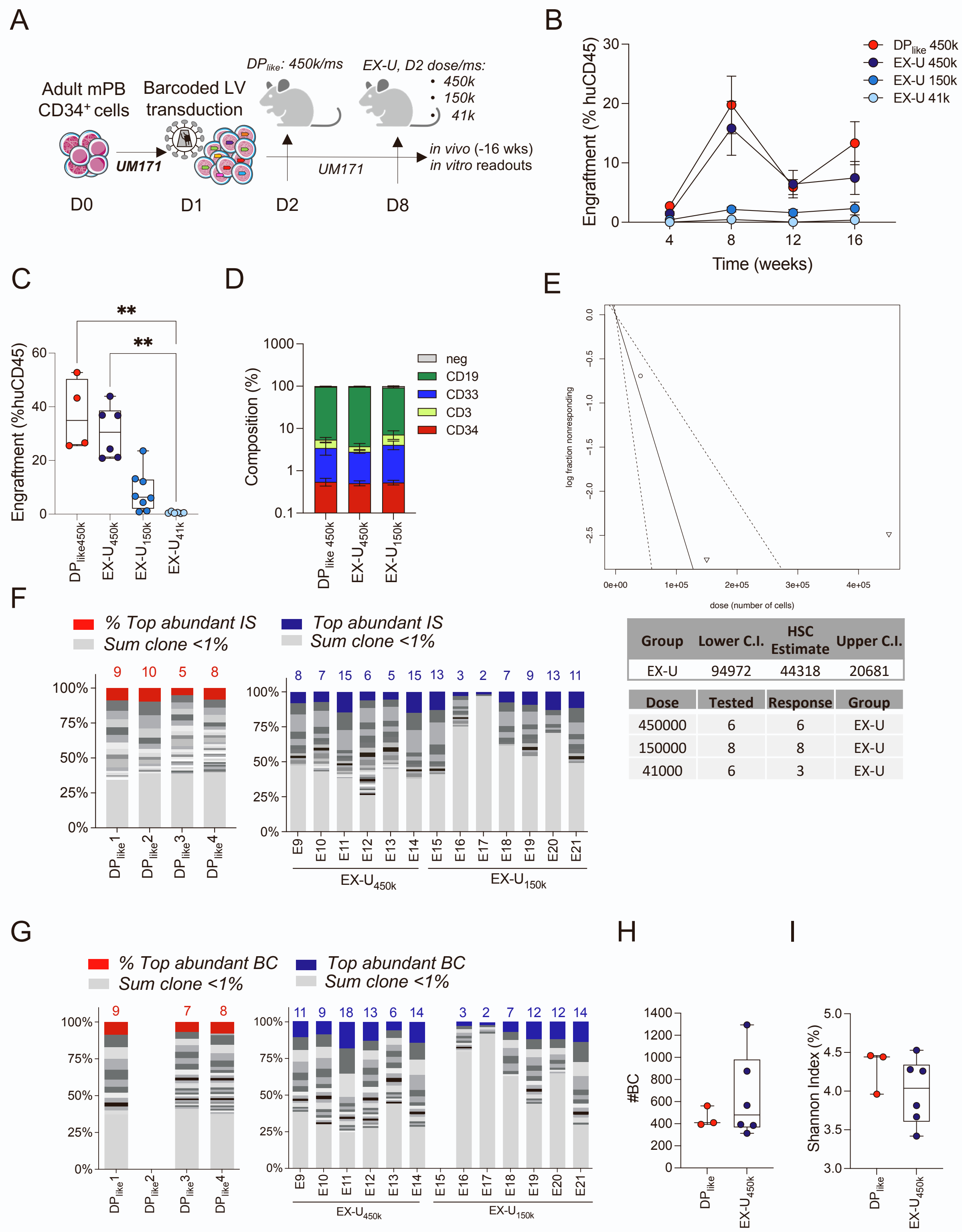

Group	Lower C.I.	HSC Estimate	Upper C.I.
EX-U	94972	44318	20681

Dose	Tested	Response	Group
450000	6	6	EX-U
150000	8	8	EX-U
41000	6	3	EX-U

Figure S1 - related to Fig.1. (**A**) Schematic representation of the experimental design. $CD34^+$ cells from the mPB of an adult healthy donor were transduced with a purified lentiviral vector expressing a highly diverse barcode and expanded in the presence of UM171 (EX-U) or not (DP_{like}). The indicated doses ($k = x10^3$) of DP_{like} and EX-U cells were xenotransplanted. (**B**) Percentage of human CD45+ cells (mean ± SEM) present in the peripheral blood (PB) of DP_{like} (n=4), $EX\text{-}U_{450k}$ (n=6), $EX\text{-}U_{150k}$ (n=8) and $EX\text{-}U_{41k}$ (n=6) at the indicated number of weeks post-transplant. (**C**) Percentage of human CD45+ cells present in the bone marrow (BM) of DP_{like} (n=4), $EX\text{-}U_{450k}$ (n=6), $EX\text{-}U_{150k}$ (n=8) and $EX\text{-}U_{41k}$ (n=6) at 16 weeks post-transplant (Kruskal-Wallis test with Dunn's multiple comparison). (**D**) Lineage composition (mean ± SEM) of the human $CD45^+$ cell graft in the BM at 16 weeks post-xenotransplantation. B cells: $CD19^+$; myeloid cells: $CD33^+$; T cells: $CD3^+$; $CD34^+$: HSPC cells; lineage negative: $CD19^-CD33^-CD3^-CD34^-$. (**E**) Top: Estimation of HSC frequency through Extreme Limiting Dilution Analysis (ELDA) transplantation dose response plots with estimated mean HSC frequency corrected on day 0 equivalent doses (full line) and 95% Wald confidence intervals (dashed lines) for tested samples. Engraftment was defined as ≥0.5% hCD45+ cells in mouse BM. Bottom: table of HSC estimates on day 0 equivalent doses with 95% Wald confidence intervals and dose-response table with number of tests and successful engraftment cases. (**F**) Relative abundance clonality analysis. Each bar represents the relative abundance of insertion sites from individual mice pertaining to the DP_{like} (left) or $EX\text{-}U_{450K}$ and $EX\text{-}U_{150K}$ groups (right). Bottom light grey stacks indicate the sum of insertions with <1% relative abundance, middle stacks indicate insertions with >1% abundance, where the top abundant insertions are highlighted in red or blue respectively. Number of retrieved top abundant insertions are reported above each column. (**G**) Same as in E, using the unique barcode as a clonal readout (Table S1). Samples MM2 and E15 did not pass prespecified quality thresholds and were discarded from downstream analysis. (**H**) Box plots indicating number of unique barcodes. Each dot represents

an individual mouse. (**I**) Box plots indicating the Shannon diversity index as measure of clonal population diversity analyzed by barcode. Each dot indicates an individual mouse of the indicated group.

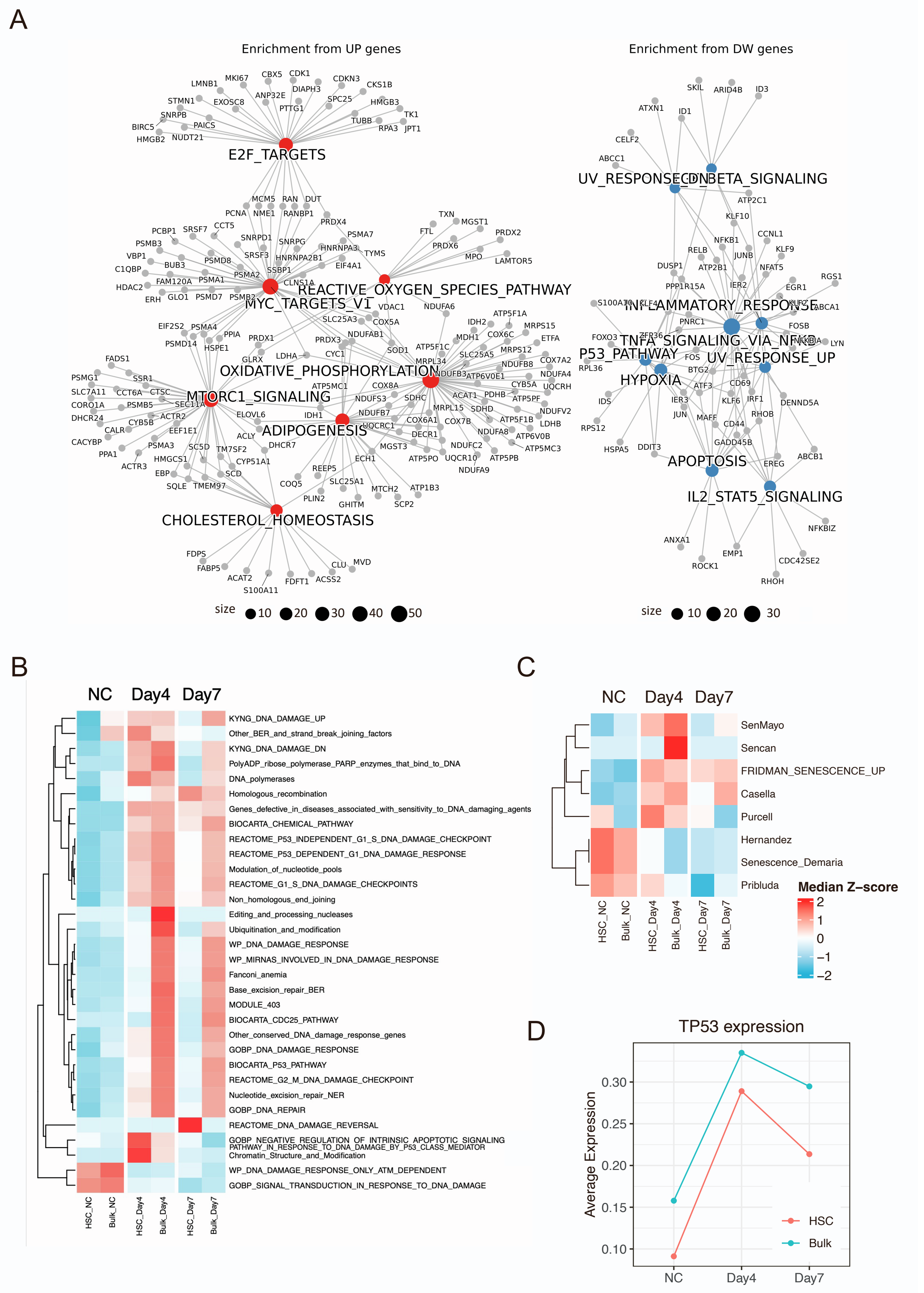
A
Enrichment from UP genes
E2F_TARGETS
MYC_TARGETS_V1
REACTIVE_OXYGEN_SPECIES_PATHWAY
OXIDATIVE_PHOSPHORYLATION
MTORC1_SIGNALING
ADIPOGENESIS
CHOLESTEROL_HOMEOSTASIS
size 10 20 30 40 50
Enrichment from DW genes
UV_RESPONSE_DN
TGF_BETA_SIGNALING
INFLAMMATORY_RESPONSE
TNFA_SIGNALING_VIA_NFKB
P53_PATHWAY
UV_RESPONSE_UP
HYPOXIA
APOPTOSIS
IL2_STAT5_SIGNALING
size 10 20 30
B
NC Day4 Day7
KYNG_DNA_DAMAGE_UP
Other_BER_and_strand_break_joining_factors
KYNG_DNA_DAMAGE_DN
PolyADP_ribose_polymerase_PARP_enzymes_that_bind_to_DNA
DNA_polymerases
Homologous_recombination
Genes_defective_in_diseases_associated_with_sensitivity_to_DNA_damaging_agents
BIOCARTA_CHEMICAL_PATHWAY
REACTOME_P53_INDEPENDENT_G1_S_DNA_DAMAGE_CHECKPOINT
REACTOME_P53_DEPENDENT_G1_DNA_DAMAGE_RESPONSE
Modulation_of_nucleotide_pools
REACTOME_G1_S_DNA_DAMAGE_CHECKPOINTS
Non_homologous_end_joining
Editing_and_processing_nucleases
Ubiquitination_and_modification
WP_DNA_DAMAGE_RESPONSE
WP_MIRNAS_INVOLVED_IN_DNA_DAMAGE_RESPONSE
Fanconi_anemia
Base_excision_repair_BER
MODULE_403
BIOCARTA_CDC25_PATHWAY
Other_conserved_DNA_damage_response_genes
GOBP_DNA_DAMAGE_RESPONSE
BIOCARTA_P53_PATHWAY
REACTOME_G2_M_DNA_DAMAGE_CHECKPOINT
Nucleotide_excision_repair_NER
GOBP_DNA_REPAIR
REACTOME_DNA_DAMAGE_REVERSAL
GOBP_NEGATIVE_REGULATION_OF_INTRINSIC_APOPTOTIC_SIGNALING_PATHWAY_IN_RESPONSE_TO_DNA_DAMAGE_BY_P53_CLASS_MEDIATOR
Chromatin_Structure_and_Modification
WP_DNA_DAMAGE_RESPONSE_ONLY_ATM_DEPENDENT
GOBP_SIGNAL_TRANSDUCTION_IN_RESPONSE_TO_DNA_DAMAGE
HSC_NC Bulk_NC HSC_Day4 Bulk_Day4 HSC_Day7 Bulk_Day7
C
NC Day4 Day7
SenMayo
Sencan
FRIDMAN_SENESCENCE_UP
Casella
Purcell
Hernandez
Senescence_Demaria
Pribluda
HSC_NC Bulk_NC HSC_Day4 Bulk_Day4 HSC_Day7 Bulk_Day7
Median Z-score
2 1 0 −1 −2
D
TP53 expression
Average Expression
0.30 0.25 0.20 0.15 0.10
NC Day4 Day7
HSC
Bulk

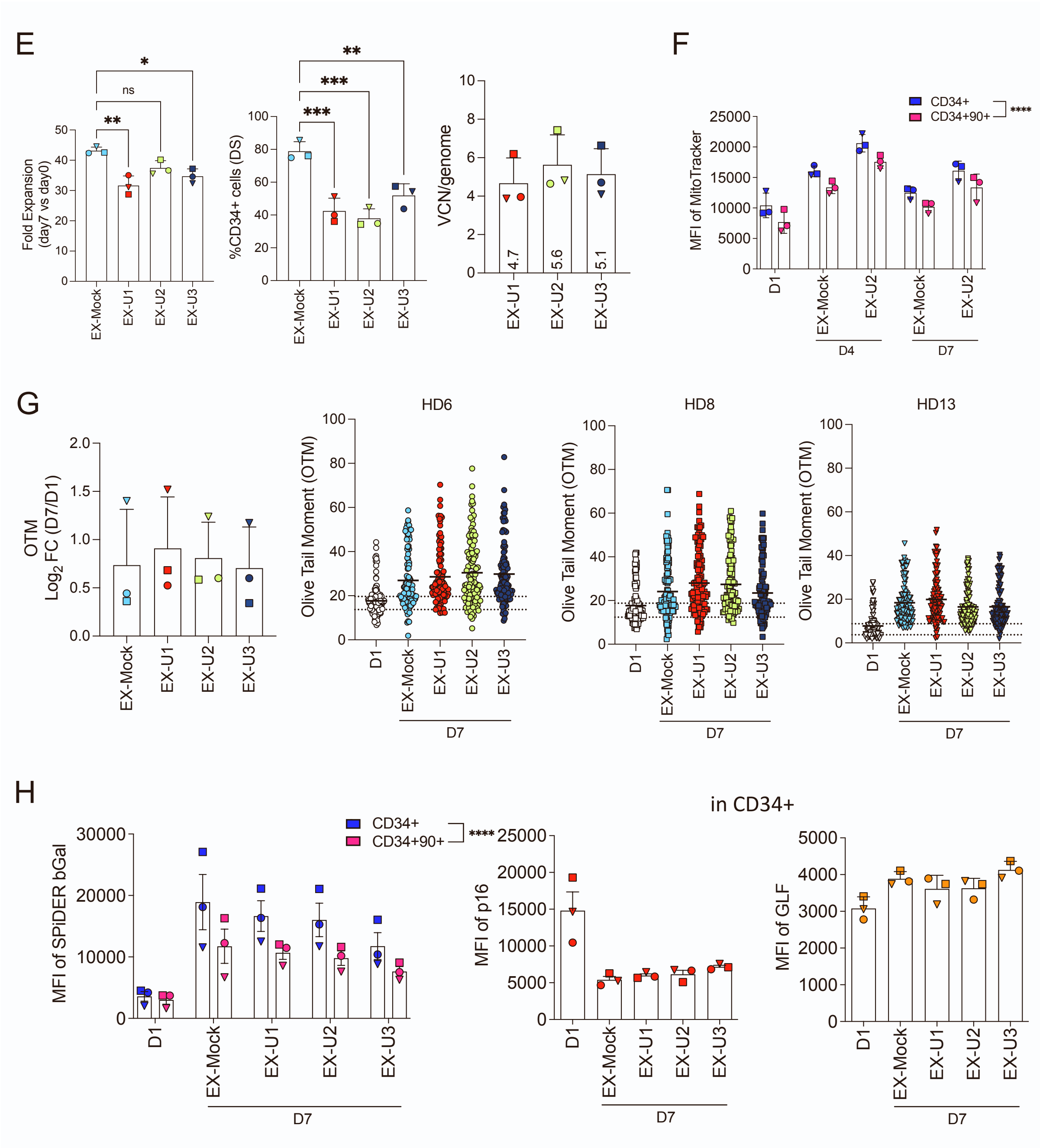

Figure S2 - related to Fig.2. (A) ORA with MSigDB hallmark pathways enriched from commonly up- or down-regulated genes over time (from uncultured to day 4 [early] or day 8 [late]) across populations. The cnetplots display enriched terms and their associated genes: upregulated genes are shown in the left plot, and downregulated genes in the right plot. Dot size indicates the number of genes associated with each term. For further details, see the scRNA-seq Methods section. (**B-C**) Heatmap of enrichment for DNA damage (B) and cellular

senescence (C) related gene signatures in HSC or bulk cell clusters either prior to ex-vivo expansion (NC: not cultured) or at different culture timepoints (D4 and D7) from the scRNAseq data described in Fig. 2C. Signatures were derived from curated reference gene lists (see Methods). (**D**) Mean single-transcript expression of *TP53* in HSC bulk (non-HSC) cell clusters prior to (NC) and at different timepoints of ex-vivo expansion (D4, D7) from the scRNAseq dataset of Fig. 2C. (**E**) In vitro expansion, immunophenotype, and transduction efficiency of mPB $CD34^+$ cells from n = 3 healthy donors cultured under EX-U conditions with lentiviral transduction at different prestimulation time windows (EX-U1: 24h, EX-U2: 36h or EX-U3: 72h) or mock (Mock). Left to right: fold expansion (vs day 0), percentage of $CD34^+$ cells at the end of culture, and vector copy number (VCN) per genome measured by ddPCR in transduced conditions. Each symbol represents one donor; bars indicate mean ± SEM. (**F**) Mitochondrial mass assessed by MitoTracker Green staining and flow cytometry in mPB $CD34^+$ cells from n=3 healthy donors at D1, D4, and D7 of EX-U culture, ±LV (EX-U2: 36h prestimulation). Geometric mean fluorescence intensity (gMFI) is shown for total $CD34^+$ cells and the $CD34^+CD90^+$ subset. Each symbol represents one donor. (**G**) DNA damage measured by alkaline COMET assay in mPB $CD34^+$ cells from n = 3 healthy donors. Left: intra-donor $\log_2$ fold change (D7/D1) of olive tail moment (OTM) across conditions. Right: single-cell OTM values at indicated time points (D1, D7) and culture conditions for each donor; each dot represents one analyzed cell. Bars indicate mean ± SEM where applicable. Dashed horizontal line represent the D1 25th and 75th percentile for each donor. (**H**) Flow cytometry–based quantification of senescence-associated markers in mPB $CD34^+$ cells from n=3 healthy donors at D1 and D7 of EX-U culture, ±LV at different prestimulation time windows (EX-U1: 24h, EX-U2: 36h or EX-U3: 72h). Left to right: senescence-associated β-galactosidase (SA-β-Gal) activity, intracellular p16, and lipofuscin (GLF) signal. SA-β-Gal is shown for both $CD34^+$ and $CD34^+CD90^+$ populations, including paired comparison between subsets; p16 and GLF are

shown for $CD34^+$ cells only. Each symbol represents one donor; bars indicate mean ± SEM. Statistical analysis, unless specified otherwise: paired Wilcoxon test.

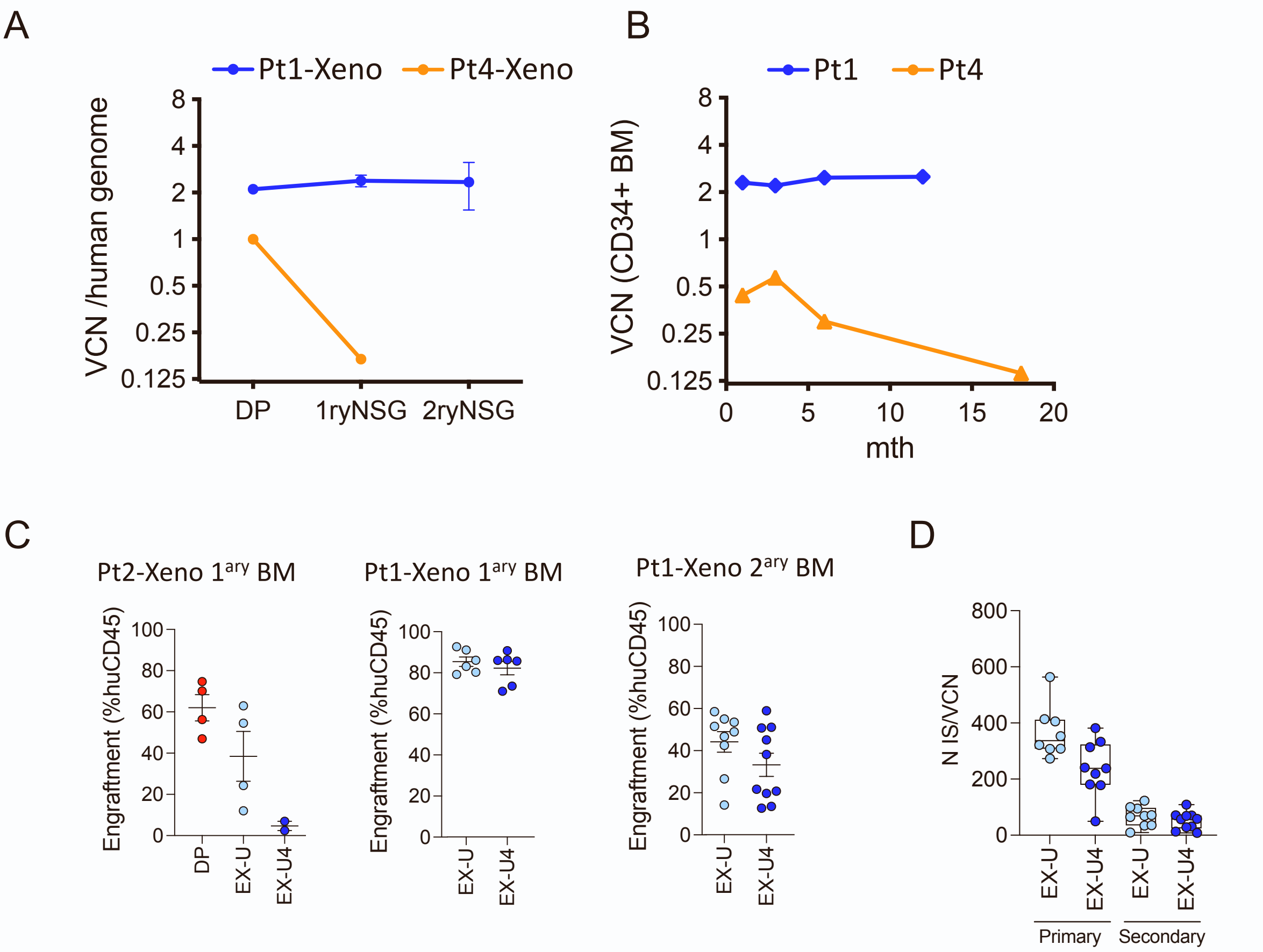

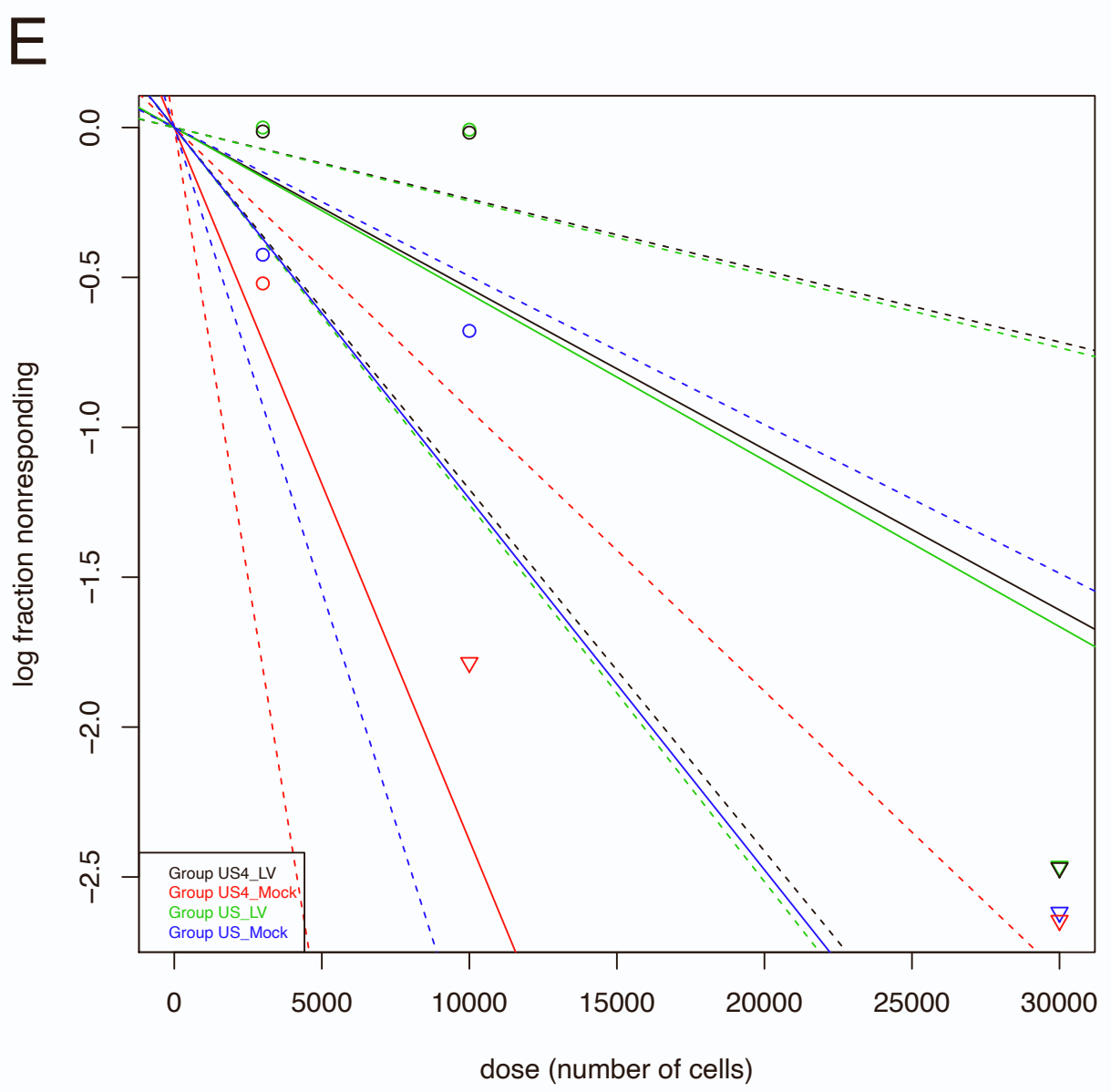

Dose	Tested	Response	Group
30000	7	7	US_Mock
10000	2	1	US_Mock
3000	3	1	US_Mock
30000	6	6	US_LV
10000	3	0	US_LV
3000	4	0	US_LV
30000	7	7	US4_Mock
10000	3	3	US4_Mock
3000	5	2	US4_Mock
30000	6	6	US4_LV
10000	3	0	US4_LV
3000	5	0	US4_LV

Group	Lower C.I.	HSC Estimate	Upper C.I.
US4_LV	41935	18640	8285
US4_Mock	10635	4206	1663
US_LV	40835	18018	7950
US_Mock	20174	8077	3234

Figure S3 - related to Fig.2 and Fig.4. (**A**) Transduction levels in the drug product (DP) (as measured by vector copy number analysis) for MPS patients Pt1 and Pt4 after 14-day in vitro culture or after xenotransplantation of the DP into primary NSG mouse recipients (1ryNSG; Pt1-Xeno: n=5 mice; Pt4-Xeno: n=8 mice) and after secondary transplantation (2ryNSG; Pt1-Xeno: n=3 mice) (mean ± SEM). (**B**) VCN in BM $CD34^+$ cells in the patients over time following gene therapy (subset of the data published in Gentner et al *(30)*, Figure S7). (**C**) Xenotransplantation of expanded DPs from MPS patients Pt1 and Pt2, expanded either in the presence of UM171 (EX-U, see Fig.1D, E) or a combination of UM171 and 4HPR (EX-U4). Human $CD45^+$ cell engraftment is shown in the bone marrow of primary (1^{ary}) recipients (Pt2-Xeno, 17 weeks; Pt1-Xeno, 12 weeks. Endpoint analysis) and secondary (2^{ary}) recipients of the human graft harvested from the bone marrow of 1^{ary} recipients from Pt1-Xeno (12 weeks endpoint). (**D**) Number of unique insertion sites normalized on vector copy number found in the BM of 1^{ary} and 2^{ary} recipient mice transplanted with the expanded DP from Pt1. See also Fig.1L). (**E**) Estimation of HSC frequency through Extreme Limiting Dilution Analysis (ELDA) transplantation dose response plots with estimated mean HSC frequency corrected on day 0 equivalent doses (full line) and 95% Wald confidence intervals (dashed lines) for tested samples. Engraftment was defined as ≥0.5% $hCD45^+$ cells in mouse BM. Bottom: table of HSC estimates on day 0 equivalent doses with 95% Wald confidence intervals and dose-response table with number of tests and successful engraftment cases.

SUPPLEMENTAL TABLES:

Table S1: LV Barcoding data shown in Fig S1F. Sheet 1 describes metadata associated with analyzed samples including group, VCN, % of engraftment and % of transduction (NGFR). This sheet is the sample sheet provided to the barcode data analysis script and includes the cutoff and MaxN variables representing the minimal % of abundance to retain a barcode and the number of maximum mice that can share a barcode within each group to be retained in the downstream analysis. Sheet 2 shows the raw data produced by barseq software in tabular format. Sheet 3 includes the filtered version of sheet 2 according to applied filters. Sheet 4 shows the complete processed data provided by the data analysis pipeline with all variables included. Sheet 5 shows the intragroup sharing percentage (1 mouse vs all intragroup). Sheet 6 summarizes the number of barcodes identified in each sample/group. Sheet 7 shows Shannon diversity indexes produced with vegan R package.

Table S2: scRNAseq data from Fig2C dataset. Sheet1 describes basic metrics including median number of UMIs and expressed genes per cell (nCount and nFeatures) as well as the % of mitochondrial genes per cell. Sheet 2 shows the cells distribution across samples and cell types, including the cell type % values within each sample used as input for generation of Fig.2C and Fig.2F. Sheet 3 includes cell type annotation according to cluster resolutions within the dataset metadata. Sheet 4 to 7 report marker genes according to different granularity of clustering and cell type annotations.

Table S3: scRNAseq data from Fig.2H dataset. describes basic metrics including median number of UMIs and expressed genes per cell (nCount and nFeatures) as well as the % of mitochondrial genes per cell. Sheet 2 shows the cells distribution across samples and cell types.

Sheet 3 and 4 show the distribution of WPRE positive and negative cells across timepoints and cell types for Pt1 and Pt4 respectively.

Table S4: LV Barcoding data from Fig. 3. Sheet 1 describes metadata associated with analyzed samples including group, VCN, % of engraftment and % of transduction (NGFR). This sheet is the sample sheet provided to the barcode data analysis script and includes the cutoff and MaxN variables representing the minimal % of abundance to retain a barcode and the number of maximum mice that can share a barcode within each group to be retained in the downstream analysis. Sheet 2 and Sheet 3 show the raw and filtered data output from barseq software. Sheet 4 shows the complete processed data provided by the data analysis pipeline with all variables included. Sheet 5 shows the intragroup sharing percentage (1 mouse vs all intragroup). Sheet 6 reports the number of barcodes identified in each sample/group. Sheet 7 shows the Shannon diversity indexes produced with vegan R package.

Table S5: Same as Table S4, comparing SCGM (EX-U) primary & secondary transplant samples and SCGM (EX-US) primary and secondary transplant samples.

Table S6: Same as Table S4, comparing transduction timing samples from DP-like-U1 to EX-U1, EX-U2 and EX-U3 groups.

Table S7: BulkRNA sequencing analysis comparing 72h vs 24h. Sheet 1 and 2 report differentially expressed genes in the two batches (1vs1). Sheet 3 and 4 report concordant up and down regulated genes from the 72h vs 24h comparison across the two different batches. Sheet 5 reports enriched terms from ORA using the reactome database. Sheet 6 shows the GSEA results from integrated batches using custom senescence reference signatures.

Table S8: For the cytokine optimization experiments (Fig. 3A–C), linear mixed-effects models were fitted using the lme4 and lmerTest R packages, with IL3 dose and IL6 dose as fixed effects (including their interaction) and donor as a random intercept. Overall effects were assessed by

Type III ANOVA with Satterthwaite's approximation for denominator degrees of freedom. Post hoc pairwise comparisons of estimated marginal means (emmeans package) were adjusted using the Holm method.
